# Mechanistic Study of Glycerol Electro-Oxidation on Ni(OH)_2_/NiOOH Electrodes

**DOI:** 10.1021/jacs.6c00726

**Published:** 2026-04-07

**Authors:** Youli Yu, Yifeng Wang, Hanzhi Ye, Sid Halder, Guangmeimei Yang, Boxi Ye, Santosh Kumar, Georg Held, James R. Durrant, Maria-Magdalena Titirici, Reshma R. Rao

**Affiliations:** † Department of Materials, 4615 Imperial College London, Exhibition Road, SW7 2AZ London, U.K.; ‡ The Grantham Institute for Climate Change, Imperial College London, Exhibition Road, SW7 2AZ London, U.K.; § Department of Chemical Engineering, Imperial College London, Exhibition Road, SW7 2AZ London, U.K.; ∥ Department of Chemistry, Imperial College London, Exhibition Road, SW7 2AZ London, U.K.; ⊥ Diamond Light Source, 120796 Harwell Science and Innovation Campus, Fermi Ave, OX11 0DE Didcot, U.K.; # Advanced Institute for Materials Research, Tohoku University, 2 Chome-1-1 Katahira, Aoba Ward, Sendai, Miyagi 980-8577, Japan

## Abstract

Glycerol oxidation reaction (GOR) is a promising valorization route to upgrade the biodiesel by-product while coproducing green hydrogen at the cathode in electrolyzers. However, the working mechanism of transition-metal-based catalysts such as Ni­(OH)_2_ remains poorly understood. Here, we employed a multioperando spectroelectrochemical approach combining UV–vis optical spectroscopy, X-ray absorption spectroscopy, and time-resolved stepped-potential spectroscopy to investigate the active oxidizing species and charge-transfer dynamics under OER and GOR conditions. We identified NiOOH (Ni^3+^) as the active species for GOR, whereas the formation of higher-valent NiOO (Ni^4+^) species is completely suppressed in the presence of glycerol. The accumulation of surface-adsorbed glycerol molecules is the rate-determining step (τ ∼ 27.9 s at 1.47 V_RHE_), occurring slower than the intrinsic catalytic step of glycerol reaction (τ ∼ 3.2 s at 1.47 V_RHE_), which involves oxidation and bond cleavage. In contrast, the kinetics of the OER are significantly slower (τ ∼ 167 s at 1.47 V_RHE_), resulting in the dominance of GOR and suppression of oxygen evolution in the presence of glycerol. The potential-independent production of formic acid during GOR follows an apparent first-order dependence on NiOOH concentration, suggesting continuous C–C bond cleavage activated by reactive *O species. These findings link oxidizing species with charge-transfer dynamics, providing insight for the rational design of Ni-based catalysts for glycerol and other biomass-derived molecule oxidations.

## Graphical abstract

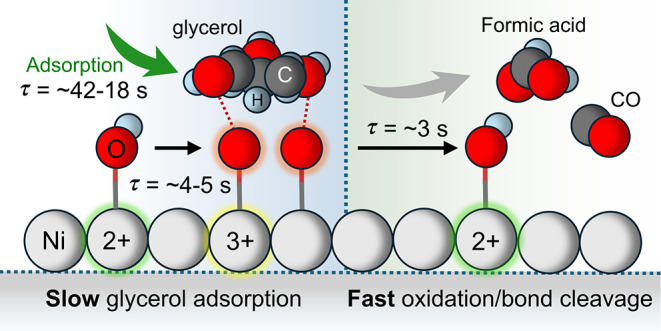

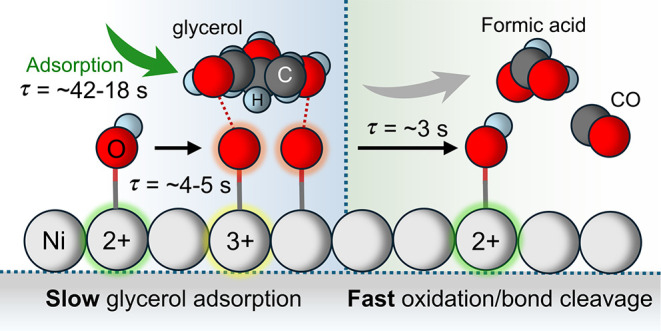

## Introduction

Electrocatalytic biomass valorization has emerged as a promising green alternative to traditional fossil-fuel-based refinery processes, which are unsustainable due to their reliance on scarce resources, high energy input, and greenhouse gas emissions.^1^ In parallel with the rapid expansion of renewable electricity and the drive for a hydrogen economy, hybrid electrolyzers have gained attention as an efficient pathway for clean chemical and fuel production.^2^ In such systems, the hydrogen evolution reaction (HER) at the cathode is coupled with the oxidation of organic molecules at the anode, substituting the oxygen evolution reaction (OER).^3–5^ While the OER is the conventional anodic reaction in aqueous electrolyte, it is intrinsically inefficient because it requires a high thermodynamic potential (1.23 V_RHE_), exhibits sluggish kinetics, leading to high electricity consumption, and produces oxygen gas, which has limited economic value.

Biomass-derived molecules, such as glycerol (GLY), have therefore attracted vast interest as alternative anodic fuels because of (1) their surplus availability as a by-product from biodiesel production (about 10 wt % of biodiesel output, priced at less than US$ 0.6 kg^–1^ for crude glycerol);^6^ (2) their potential for producing value-added chemicals such as formic acid (C1), glycolic acid (C2), and glyceric acid (C3), which have wide range of applications in the pharmaceutical, cosmetic, and fine chemicals industries;^7,8^ as well as (3) their low thermodynamic potential for the first deprotonation step (0–0.4 V_RHE_).^9–11^ Current state-of-the-art catalysts for the glycerol oxidation reaction (GOR) still largely rely on precious metal catalysts (Pt, Au, Pd, etc.) because of their ability to produce C3/C2 products at low operating potential.^12–16^ However, the disadvantages of high capital cost, greenhouse gas emission and environmental damage associated with upstream mining and ore processing (including energy-intensive extraction, toxic tailings, and land disturbance), as well as active site poisoning, and low selectivity toward a single product limit their scalability.^17–19^

Among the cheaper alternatives, Ni, especially its hydroxides, has shown promise for production of green fuels and chemicals, initially in alkaline electrolyzers,^20–23^ and more recently in alkaline organic oxidation.^24–26^ Reactive oxyhydroxide intermediates that form under oxidizing potentials can be beneficial for alcohol oxidation, such as GOR.^27–31^ Recent studies have also demonstrated that combining Ni with precious metals is effective in tuning the selectivity toward C2/C3 products (e.g., lactic acid and glycolic acid) and reducing site poisoning.^31,32^ Luo et al. combined optical UV–vis spectroscopy and X-ray absorption spectroscopy to study PtNi alloys for glycerol oxidation.^33^ This work speculates that Ni itself does not directly participate in the reaction but rather modulates the electronic structure of Pt in the bimetallic system. Another study found that for the electrodeposited porous Ni­(OH)_2_-supported Au catalytic system, the performance boost originated from the formation of an *OH-rich environment provided by Ni­(OH)_2_.^34^

However, the true working mechanism for Ni itself as a catalyst for GOR has received limited attention,^35–40^ inhibiting more effective strategies for designing a better Ni-based system. As early as the 1970s, the indirect mechanism was proposed by Fleishmann et al. In this mechanism, the Ni­(OH)_2_/NiOOH couple acts as a redox mediator; the NiOOH generated under applied potential can partake in a rate-determining chemical step for glycerol oxidation.^41^ However, later studies proposed that higher valence forms of nickel oxyhydroxide species (denoted as NiOO (Ni^4+^) in this study) also contribute to GOR via a hydride transfer mechanism,^42–44^ while other studies argued that the Ni^4+^ species can trigger the competing OER.^45,46^ A recent study using spectroelectrochemical (optical UV–vis) technique demonstrated an increase in absorption at ∼600 nm in the presence of glycerol compared to the OER-only condition, suggesting different species are involved in GOR and OER.^47^ The ambiguity of assigning the active oxidizing species arises from the coexistence of multiple oxidizing species during the electrochemical oxidation process.^48–51^ Hence, a spectroelectrochemical study capable of deconvoluting and assigning the oxidizing species as well as revealing the interfacial reactivity under working conditions is needed.

Our group has published several studies using *operando* spectroscopy methods to deconvolute the redox changes and quantitatively evaluate kinetic constants.^48–50,52–55^ Herein, a similar experimental approach was followed: we combined *operando* spectroelectrochemical techniques (optical UV–vis, Ni L-edge, O K-edge X-ray absorption spectroscopy) to identify and quantify the active oxidizing species during the OER and GOR. NiOOH (Ni^3+^) was identified to be the active oxidizing species for GOR. Time-resolved optical UV–vis spectroscopy was used to experimentally measure the time constants for different processes occurring at the catalytic site, including oxidation of the active site and the catalytic turnover rate. The rate-determining step for GOR was found to be the accumulation of surface-adsorbed glycerol molecules (τ ∼ 27.9 s at 1.47 V_RHE_), which is slower than both the accumulation of active oxidizing species (Ni^3+^) (τ ∼ 4.3 s at 1.47 V_RHE_) and the catalytic step of glycerol oxidation (τ ∼ 3 s). This is in stark contrast to OER, where the reaction rate is τ ∼ 165 s at 1.47 V_RHE_, 2 orders of magnitude slower than the accumulation of oxidizing species (Ni^4+^) (τ ∼ 2 s). Moreover, we found that the GOR exhibits a first-order dependence on the concentration of oxidized species in the studied potential window (1.4–1.58 V_RHE_). By combining spectroscopic insights with product analysis, including online electrochemical mass spectrometry (EC-MS) results, we propose a reaction mechanism to account for the potential-independent formic acid production and complete suppression of water oxidation in the presence of glycerol. Therefore, using multimodal spectroscopy, we experimentally determine the rate-determining step of the glycerol oxidation of Ni-based catalysts, which provides new insights into the design of more efficient Ni-based catalysts for GOR.

## Results and Discussion

### Identifying the Active Species for Glycerol Oxidation

Ni­(OH)_2_ electrodes were prepared via electrodeposition on FTO substrates following Trotochaud et al.’s protocol.^20^ Scanning electron microscopy (SEM, Figure S1) images showed a sheet-like structure. Stylus profilometry (Figure S2­(a)) gives Ra = 26 nm with a film thickness of ∼120 nm, indicating a rough surface. X-ray photoelectron spectroscopy (XPS) indicates a mixed Ni^2+^/Ni^3+^ surface state in the as-prepared film (Figure S3). In contrast, Ni K-edge X-ray absorption near-edge structure (XANES) is dominated by the Ni^2+^ state (Figure S4­(a,b)), consistent with the greater bulk sensitivity of XANES and suggesting that the Ni^3+^ fraction is relatively minor and/or concentrated near the surface (e.g., due to partial surface oxidation upon air exposure). The sample exhibits low crystallinity, and extended X-ray absorption fine structure (EXAFS, Figure S4­(c)) shows predominantly short-range order with a weak signal from higher-shell contributions. X-ray diffraction (XRD,Figure S5) further indicated no distinct Bragg reflections apart from the substrate FTO peaks and only a broad diffuse feature. Overall, the material characterizations showed good consistency with previous studies on electrodeposited Ni­(OH)_2_.^20,43^

The electrochemical performance was measured in 0.1 M KOH and 0.1 M KOH + 0.1 M glycerol conditions (iron-free). XPS of the working electrodes after cycling using a Pt counter electrode showed no detectable Pt redeposition signal within the XPS detection limit (Figure S6). Figure 1(a) shows the linear sweep voltammograms in 0.1 M KOH (red) and 0.1 M KOH + 0.1 M glycerol (blue). Upon increasing the potential to 1.58 V_RHE_, markedly different activity can be observed between the linear sweep voltammograms with and without glycerol. An appreciable current (0.13 mA/cm^2^) for GOR can be detected at ∼1.4 V_RHE_, which is nearly 100 mV lower than the OER. Furthermore, the redox feature at 1.34–1.39 V_RHE_ in 0.1 M KOH is not evident after adding glycerol to the electrolyte, suggesting differences in redox kinetics in the presence of glycerol.

**Figure 1:**
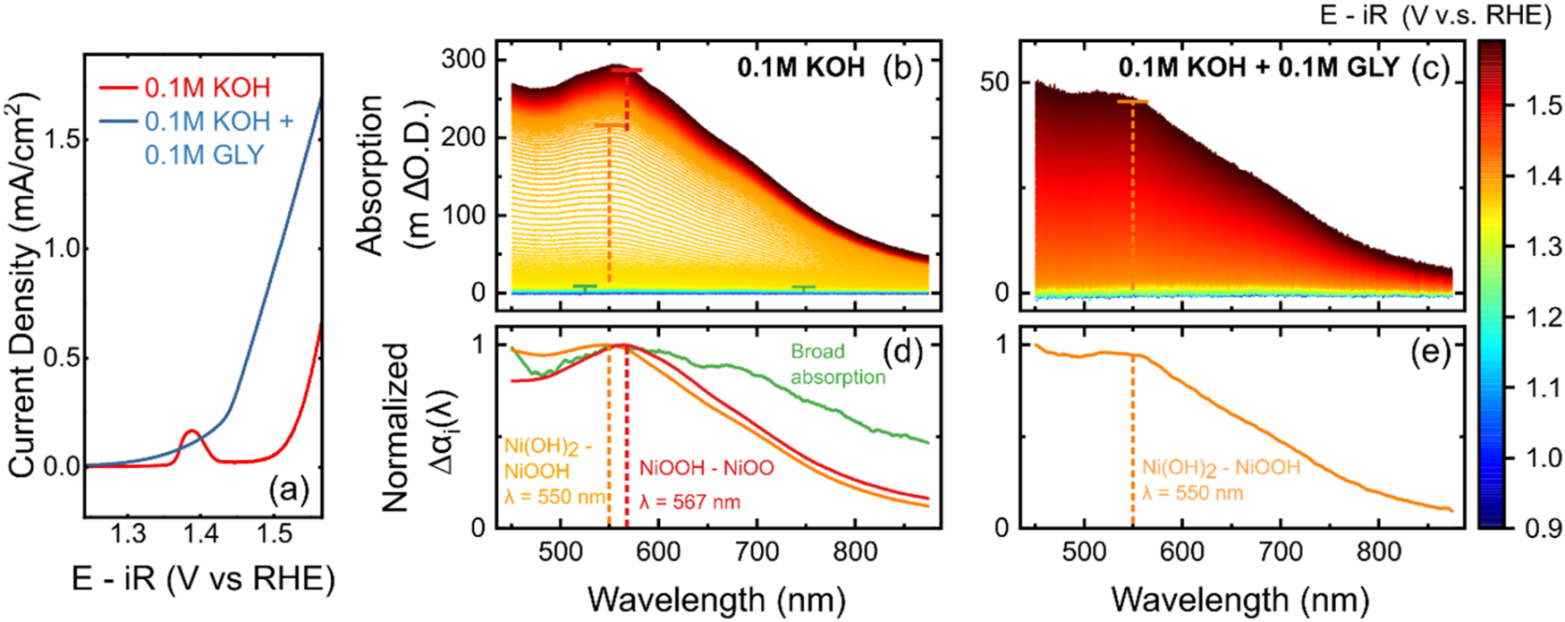
(a) Measured current density in iron-free 0.1 M KOH (red) and 0.1 M KOH + 0.1 M GLY (blue). The current density was recorded along with the optical spectra. Differential absorption spectra of Ni­(OH)_2_ against the starting potential spectrum (0.91 V_RHE_) in (b) 0.1 M KOH and (c) 0.1 M KOH + 0.1 M GLY. The normalized differential component absorption spectra for (d) three individual redox transitions at 1.20–1.30 V_RHE_ (green), 1.34–1.39 V_RHE_ (orange), and 1.40–1.55 V_RHE_ (red) in 0.1 M KOH and (e) one redox transition from 1.43 to 1.70 V_RHE_ (orange) in 0.1 M KOH + 0.1 M GLY. Both spectra were scanned at 1 mV s^–1^ scan rate. A Pt coil was used as a counter electrode, and Hg/HgO (filled with 0.1 M KOH) was used as the reference electrode.

Optical UV–vis spectroscopy was used to probe potential-dependent Ni oxidation and the density of redox active sites. The optical UV–vis absorption spectra, as a function of potential, with and without glycerol, are shown in Figure 1(b,c). To deconvolute the absorption spectra into distinctive redox components, we performed differential analysis. Specifically, differential absorption spectra were extracted at 20 mV intervals by subtracting each spectrum from its neighbor (Figures S7 and S8). The spectral feature for each redox component should remain unique and consistent across the determined redox potential window after intensity normalization. The normalized differential component spectra for the data obtained in 0.1 M KOH, shown in Figure 1(d), depict the evolution of three unique spectral shapes as a function of potential, consistent with our previous work.^48,49,53,56^ Specifically, at potentials <1.3 V_RHE_, we observe a broad absorption feature, which has been assigned to structural defect oxidation, as discussed in previous work.^48^ Between potentials of 1.34 V_RHE_ and 1.39 V_RHE_, a feature with maximum absorption at ∼550 nm was identified. Finally, at potentials above 1.4 V_RHE_, a distinct absorption feature was observed, which was red-shifted by 17 nm to ∼567 nm. These latter two redox transitions have been attributed to the successive oxidation of Ni sites.^53,56^

Once the electrolyte was replaced with 0.1 M KOH + 0.1 M glycerol, only one distinct absorption feature was observed, as shown in the normalized differential absorption spectra (Figure 1(e)). The absence of the first redox spectral feature, previously attributed to structural defect oxidation, is likely due to the irreversible elimination of defect sites during the earlier measurement in 0.1 M KOH. The spectral shape corresponding to the single redox transition detected in the glycerol-containing electrolyte is similar to the second redox transition identified in 0.1 M KOH. These results suggest that further oxidation of Ni at high potentials is suppressed in the presence of glycerol. Furthermore, a comparison of the optical density corresponding to this redox transition at the absorption maximum (550 nm) revealed that the absorption decreases to ∼50 mΔO.D. in the presence of glycerol, compared to nearly 175 mΔO.D in its absence. This decrease in the density of oxidized Ni centers in the presence of glycerol may arise from (i) the faster consumption of the oxidized species during glycerol oxidation and/or (ii) partial surface coverage/site blocking by glycerol (or reaction intermediates), which reduces the population of optically observed oxidized Ni species at a given potential.

The density of redox active species (represented as an area density in Figure 2) as a function of potential can be determined by performing linear combination fitting (Figures S9 and 10, with fitting residuals within 5%) for each identified redox transition, assuming the optical absorbance is a linear sum of each redox transition’s absorbance following the Beer–Lambert law.^50^ A stepped-potential analysis (Figures S11 and S12) to experimentally translate the optical absorption to the density of oxidized species (Figure S13) was performed. The charge passed upon applying the stepped potential was correlated with the change in optical absorption under the assumption that the formation of each oxidized species is associated with a single electron transfer. The potential dependence of the redox transitions observed in 0.1 M KOH (Figure 2(a)) agrees well with reported literature on similar materials.^53,56^ The first redox transition feature will not be discussed due to the low contribution in the optical signal (∼10 mΔO.D.). The second redox transition obtained from the optical spectroscopy is at 1.34 V_RHE_, which is consistent with the redox peak position observed in the cyclic voltammogram at 1.34 V_RHE_. Following this, a final redox transition is observed prior to the evolution of oxygen at more anodic potentials. In the 0.1 M KOH + 0.1 M GLY electrolyte (Figure 2(b)), the onset of the first Ni redox is shifted to ∼1.4 V_RHE_, which is ∼0.06 V more anodic than the corresponding redox transition in 0.1 M KOH. Furthermore, the redox transition occurs over a wider potential window in glycerol-containing electrolyte, suggesting that the interaction of glycerol with the catalyst alters both the energetics and kinetics of the Ni oxidation, and substantially prevents its successive oxidation at more anodic potentials. Notably, in our previous work on PtNi alloys,^33^ we observed a complete suppression of Ni oxidation in the presence of glycerol, which we attributed to strong glycerol adsorption onto the Ni­(OH)_2_ islands, preventing further oxidation.

**Figure 2:**
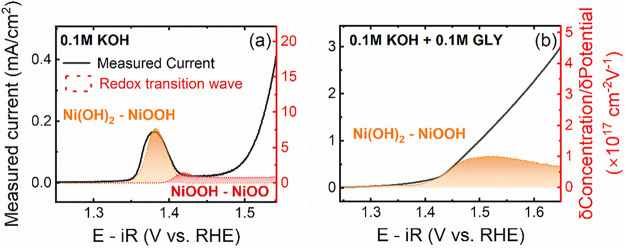
Measured linear scan voltammetry current density at 1 mV s^–1^ (left *y*-axis) and the derivatives of the optically determined redox transition densities, represent the redox transition wave (right *y*-axis) in (a) 0.1 M KOH and (b) 0.1 M KOH + 0.1 M GLY. A Pt coil was used as the counter electrode, and Hg/HgO (0.1 M KOH) was used as the reference electrode.

To determine the nature of these oxidized species detected using optical spectroscopy, *operando* near-edge X-ray absorption fine structure (NEXAFS, Ni L-edge and O K-edge) in total fluorescence yield (TFY) mode was used to track the oxidation states, as shown in Figure 3. It is worth noting that NEXAFS in the TFY mode is a bulk-sensitive technique.^74^ The amorphous Ni­(OH)_2_ film used for this study is porous (Figures S1 and S2) and volume active, where the majority of the Ni species are active,^20,57^ which thus reduces the complexity of interpreting signals from mixed phases.^58^ The formation of NiOOH (Ni^3+^) from Ni­(OH)_2_ (Ni^2+^) can be distinguished qualitatively by comparing the intensity ratio between the two absorption features of Ni L_3_ or L_2_ edges as well as the appearance of satellite peak broadening at 854.6 and 871.1 eV, as seen from the reference spectra of Ni­(OH)_2_ (Ni^2+^) and LiNiO_2_ (Ni^3+^) (Figure S14). However, due to the electrochemically unstable nature of NiOO (Ni^4+^) species, direct measurement from its standard reference sample is challenging.^59^ Zheng et al. demonstrated a way to separate the Ni L-edge of Ni^4+^ species by subtracting the fitted Ni^2+^ signal from the potassium nickel­(IV) paraperiodat K_2_Ni­(H_2_IO_6_)_2_ (Ni^4+^) reference sample’s signal, showing a positive ∼2.6 eV shift in both L_3_ and L_2_ peaks compared to the Ni^2+^ signal.^60^ From the OCP to 1.31 V_RHE_, in both 0.1 M KOH and 0.1 M KOH + 0.1 M glycerol electrolytes, Ni­(OH)_2_ spectral features remained unchanged. This aligns with our assignment of the first redox transition, detected in 0.1 M KOH at potentials <1.3 V_RHE_ for oxidation of minority defect sites. However, at 1.41 V_RHE_ and above, significant spectral changes were observed in both the Ni L–edge and the O K-edge. Specifically, at 1.41 V_RHE_ without glycerol, the Ni L_3_-edge exhibited a shift of 0.3 eV, with the predominant peak now at 854 eV, accompanied by a shoulder at 851.7 eV, as shown in Figure 3(a), suggesting the onset of the formation of Ni^4+^. The shoulder peak disappeared at 1.51 V_RHE_, indicating the full transition of the catalyst to Ni^4+^. The Ni L_2_-edge evolved from a multiplet feature to a broad peak at 871 eV, with a shift of 1.2 eV. These spectral changes are consistent with the oxidation of Ni^2+^ to Ni^4+^.^60–62^ The O K-edge spectrum (Figure 3(b)) additionally revealed a new pre-edge feature at 528.2 eV, further confirming the formation of the oxidized Ni phase. In contrast, for the 0.1 M KOH + 0.1 M glycerol electrolyte, the spectral feature of Ni^4+^ oxidation states was absent above 1.41 V_RHE_ (Figure 3(c)), with no peak appearing at 854 eV. Instead, broadening of the satellite peaks at 854.6 eV (Ni L_3_) and 871.1 eV (Ni L_2_) was observed, and the intensity ratio between the two absorption features at Ni L_3_ changed such that the absorption at 853.6 eV became dominant. Furthermore, no obvious changes were observed in the O K-edge (Figure 3(d)). Therefore, both the Ni L edge and the O K-edge data confirm NiOOH (Ni^3+^) formation rather than the progression to NiOO (Ni^4+^) in glycerol-containing electrolyte. These results align with the optical spectroscopy data and further confirm that Ni^4+^ formation is suppressed in the presence of glycerol.

**Figure 3:**
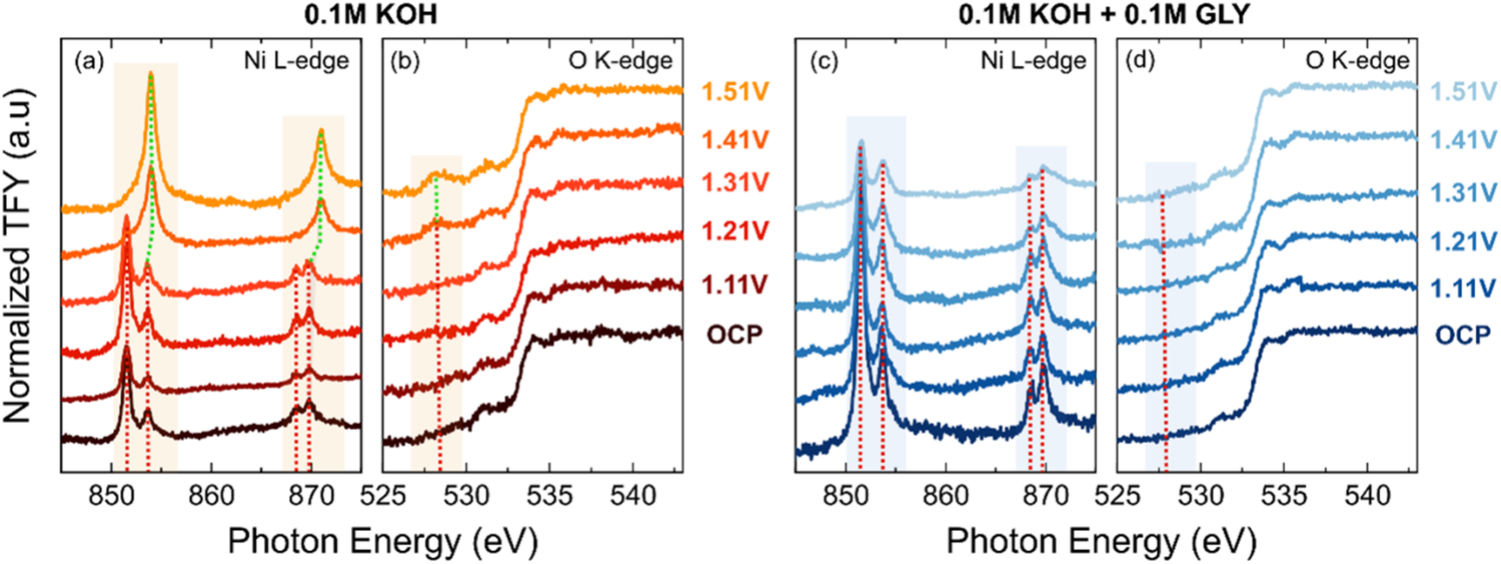
Operando Ni L-edge and O K-edge near-edge X-ray absorption fine structure (NEXAFS) spectra of electrodeposited Ni­(OH)_2_ on SiN_*x*_ membrane, measured under the total fluorescence yield (TFY) mode. Spectra were recorded from non-*iR* corrected open-circuit potential (OCP) to 1.51 V_RHE_ in (a) Ni L-edge and (b) O K-edge in Ar-saturated 0.1 M KOH; (c) Ni L-edge and (d) O K-edge in Ar-saturated 0.1 M KOH + 0.1 M GLY. The measurements were taken after holding at each potential for 10 min. A Pt rod was used as the counter electrode, and Ag/AgCl (sat. KCl) was used as the reference electrode.

### Time Constant for the Generation of Redox Active Species and Catalysis

Time-resolved optical UV–vis spectroscopy was used to study the temporal kinetics of different charge-transfer processes to determine the factors controlling the kinetics of glycerol oxidation. Specifically, the kinetic time constants (τ) were extracted and compared for different charge-transfer processes:

(1) **Accumulation of active oxidized species:** The potential was first held for 20 s in a region where no active species for OER or GOR are generated, then stepped to a higher potential where oxidizing species begin to accumulate, as shown in Figure 4(a,b red shaded region). An increase in optical absorption at the absorption maximum of each redox transition (550 nm for the GOR condition and 567 nm for the OER condition, as identified in Figure 1(b,c)) is observed upon increasing the potential, and the rate of increase depicts the kinetics of how fast the oxidizing species are accumulating at the surface. For the oxidative potential step in glycerol, a biphasic response was observed, with a fast oxidation potential-independent component followed by a potential-dependent slower component. A biphasic exponential fitting was applied, where two kinetic terms are summed^63^

$$y\left(t\right)=A_{1}\left(1-e^{-x/\tau_{1}}\right)+A_{2}\left(1-e^{-x/\tau_{2}}\right)$$

where *A*_1_ and *A*_2_ are the amplitudes (constrained to sum up to 1 after normalization), whose magnitudes represent the fractional contribution of each kinetic term; τ_1_ and τ_2_ are the time constants for the two kinetic terms. The parameters are fitted by a damped least-squares algorithm. However, due to the negligible fractional contribution (<5%) from the second kinetic term under OER-only conditions, a monophasic exponential fitting is numerically accurate enough for the fitting.

(2) **Reaction kinetics:** After the density of oxidized species reached steady state (40 s for OER-only condition and 80 s for GOR condition), the applied potential was switched to open-circuit potential (OCP). A concomitant decay of the optical signal was observed, as shown in Figure 4(a,b green shaded region). The initial rate of decay provides insight into the intrinsic reaction time constant, as demonstrated by our previous work.^50,53,64^ Briefly, the time constant is derived from linear fitting of the normalized optical signal in the time window (∼1–1.5 s), where the OCP decay is approximately linear.

(3) **Reductive discharge of oxidized species:** Instead of switching to OCP, the potential was stepped back to the region where no active species are stabilized. The optical signal intensity decreases upon application of a lower potential, and the time constant for this decrease represents the rate of reductive discharge of the oxidized species (Figure 4(c,d), blue shaded region). A monophasic exponential fitting was used because of the negligible contribution from a second kinetic term. Detailed fitting results can be found in Figures S15 and S16 and Tables S1 and S2.

For measurements in a 0.1 M KOH electrolyte (Figure 4(e)), the initial potential step was chosen to be 1.39 V_RHE_ to avoid signals from the second transition Ni­(OH)_2_ to NiOOH and only detect kinetics of NiOO (Ni^4+^) formation. NiOO (Ni^4+^) accumulation occurred rapidly on the order of ∼2 s. In contrast, the OCP decay, which reflects the intrinsic OER kinetics, was much slower, showing a strong potential dependence from ∼165 s at 1.47 V_RHE_, accelerating to ∼35 s at 1.57 V_RHE_. Our previous work has demonstrated that this decrease in time constant with increasing potential stems from multisite interaction between the oxidized species formed with increasing potentials, which drives water oxidation.^48,51^ In the presence of glycerol, the kinetic behavior changed significantly (Figure 4(f)). The accumulation of the oxidizing species shows two kinetic terms ranging from ∼4.3 s to ∼41.8 s at 1.45 V_RHE_, implying the physical presence of two charge-transfer processes. Specifically, the first fast kinetic term (τ_fast_ ∼4.3 s) is assigned to the accumulation of the active oxidized NiOOH species. Similar potential-independent time constants have been found for Ni oxidation in our previous work.^53^ Furthermore, upon applying a reductive potential step, the time constant for reduction of oxidized Ni^3+^ states was observed to be ∼2 s, suggesting no significant charge-transfer limitations in the presence of glycerol. The second slower kinetic term (τ_slow_ ∼ 18–42 s) is only present in glycerol-containing electrolyte and is strongly potential-dependent. Based on this, we attribute τ_slow_ to an adsorption-related process requiring mass transport of glycerol to the surface and binding to the active site. Hence, the physical interpretation of the two kinetic terms can be assigned to be the fast accumulation of the active NiOOH (Ni^3+^) oxidizing species and the slow adsorption of glycerol molecules, respectively.

**Figure 4:**
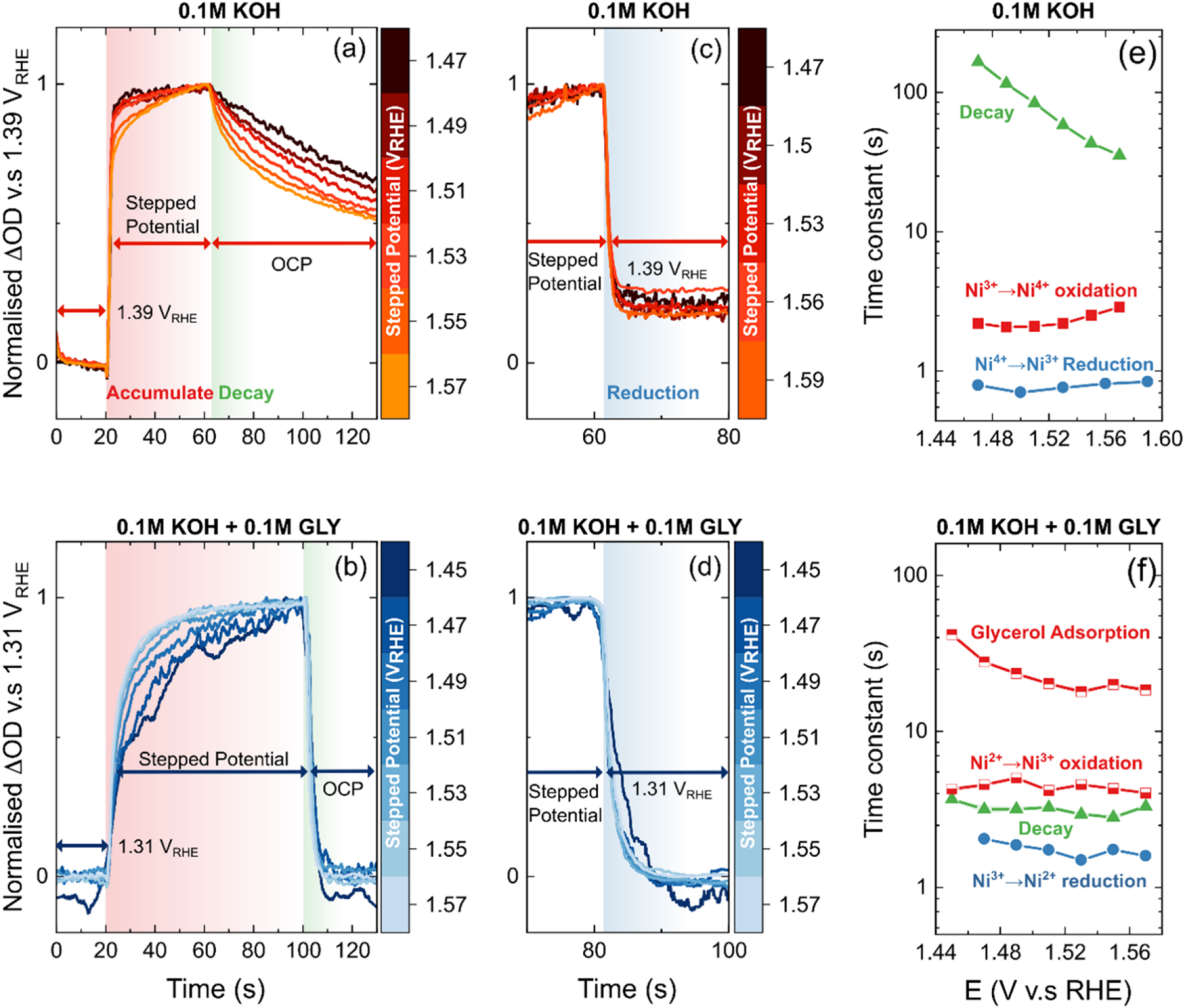
Normalized time-resolved optical response and the corresponding time constants. Normalized optical signal recorded at 567 nm under the 0.1 M KOH condition for (a) OCP decay from 1.39 V_RHE_ to applied potential and back to OCP and (b) a stepped potential from 1.39 V_RHE_ to applied potential and back to 1.39 V_RHE_. Normalized optical signal recorded at 550 nm under 0.1 M KOH + 0.1 M glycerol condition for (c) OCP decay from 1.31 V_RHE_ to applied potentials and back to OCP and (d) stepped potential from 1.31 V_RHE_ to applied potentials and back to 1.31 V_RHE_. Time constants of different charge dynamic processes are summarized (e) for the 0.1 M KOH condition and (f) for the 0.1 M KOH + 0.1 M GLY condition. The time resolution of the equipment is 30 ms.

On the contrary, the OCP decay time constant, indicative of the intrinsic GOR rate, is potential independent at ∼3 s and is comparable to the rate of oxidized NiOOH (Ni^3+^) accumulation. The potential independence of the intrinsic rate suggests that GOR is first order with respect to the oxidized NiOOH (Ni^3+^) states. Interestingly, the rate of reductive discharge is comparable to GOR kinetics (∼1–3 s) and faster than the rate of NiOOH accumulation. Based on these results, we thus hypothesize that the rate-determining step for glycerol oxidation is the accumulation of surface-adsorbed glycerol molecules, while the chemical oxidation between the adsorbed glycerol molecules and NiOOH is significantly faster.

### Proposed Reaction Mechanism

To understand how redox and reaction kinetics influence GOR product formation, high performance liquid chromatography (HPLC) and electrochemical mass spectrometry (EC-MS) were combined to provide a comprehensive analysis of the generated reaction products. In agreement with previous reports on a Ni-based system,^65–69^ formic acid was identified as the only liquid product by HPLC in this study. However, the minor presence of other liquid products cannot be excluded due to the low conversion rate and the HPLC detection limit (∼1 mM, as indicated by the calibration curve in Figure S17). To assess the effect of the counter electrodes, the electrochemical performance using a Pt coil and a graphite rod was compared (Figures S18 and S19), and no meaningful differences were observed within experimental uncertainty. For product quantification, the HPLC samples were prepared on carbon paper substrates, in contrast to the FTO used in optical UV–vis spectroscopy measurements, to ensure higher catalytic activity suitable for HPLC quantification. As shown in Figure S19­(b), the larger geometric surface area and lower internal resistance of carbon paper result in higher geometric current densities.^46^ Four potentials (1.36, 1.41, 1.46, and 1.51 V_RHE_) were selected for study, and the corresponding chronoamperometry can be found in Figure S19­(c). Interestingly, the Faradic efficiency of producing formic acid varies from ∼70–90% (Figure 5(a)) but showed no clear dependency on the applied potential and the holding time (one or two hours).

Complementary EC-MS analysis revealed minor gaseous signal at *m*/*z* = 28 under anodic potentials (Figure 5(b) left), the glassy carbon substrate was found to be stable under glycerol-containing condition (Figure S20). Here, we assign the *m*/*z* = 28 signal to be gaseous CO rather than N_2_, since the only possible nitrogen source can come from the deposition precursor (Ni­(NO_3_)_2_), and no such signal was observed under 0.1 M KOH alone. It is worth noting that no CO_2_ signal was observed within the detection limit of pmol,^70^ which may reflect either limited formation of gaseous CO_2_ under these conditions or retention of CO_2_ in the alkaline electrolyte as carbonate, thereby eliminating the gas-phase detection by EC-MS. Although the quantitative analysis of the CO production was not evaluated, its presence could partially explain why the Faradic efficiency for liquid products did not reach 100%. More importantly, negligible O_2_ was detected in the presence of glycerol within the pmol detection limit, providing direct evidence that the OER is effectively suppressed under GOR conditions. This is also consistent with the absent identification of the oxidized NiOO (Ni^4+^) species in the presence of glycerol, which has been widely proposed to be the active sites for OER.^71^

From the analyses of kinetic time constants and complementary product analysis, we conclude that once NiOOH is formed, the dehydrogenation, subsequent oxidation, and C–C bond cleavage of glycerol occur spontaneously. However, in contrast to the previously proposed indirect mechanism, which considers the chemical oxidation of adsorbed glycerol as RDS,^41^ or to other proposed oxidation pathways involving NiOO (Ni^4+^) as the active species,^43^ we proposed that (1) the rate-determining step for GOR is the accumulation of surface-adsorbed glycerol molecules, and (2) the formation of NiOO (Ni^4+^) species is completely suppressed.

Specifically, the OCP decays exhibit nearly the same rate (∼1–3 s) under GOR conditions, independent of potential. This is consistent with an apparent first-order reaction characteristic, suggesting that the density of the NiOOH species directly determines the rate of the reaction. This finding is further supported by a rate-law analysis (Figure S21), where the deconvoluted glycerol oxidation current showed a nearly first-order dependence on the concentration of the active oxidizing species (NiOOH) under both 1 mv s^–1^ LSV and the steady-state condition, i.e., rate of glycerol oxidation = k*­[NiOOH], where *k* is the reaction rate constant independent of potential. Furthermore, previous studies employing in situ ATR-SEIRAS as well as DFT calculation on a similar Ni­(OH)_2_ system suggest a pathway in which glycerol is first converted to glyceraldehyde (C3), then cleaved and oxidized to glyceric and glycolic acid (C2), followed by complete oxidation to formic acid and gaseous species (C1), before desorption from the surface.^72^ The proposed reaction pathway aligns well with our observations, where potential-independent formation of formic acid and CO (C1) was detected.^73^ From the kinetic standpoint, the OCP decay rate decreases by nearly an order of magnitude, from ∼35 s in the absence of glycerol to ∼3.3 s in the presence of glycerol at 1.57 V_RHE_. This contrast in reaction kinetics, coupled with the absence of more oxidized states in the presence of glycerol, also explains the dominance of GOR over OER at comparable potentials. Glycerol rapidly reacts with the available *O sites via a first-order reaction pathway, before the molecular oxygen release can occur, where the coupling of metal-oxo species (O–O bond) formation relies on the interaction between multiple oxidized states.^48^

Based on the mechanistic understanding and proposed reaction pathway, the drastic difference in selectivity between Ni hydroxides and other precious-metal-based catalysts can be attributed to the continuous supply of active *O species at anodic potentials. These *O species can promote C–H activation at the surface, leading to spontaneous oxidation and C–C bond cleavage. Consequently, the C–C bond cleavage is more likely to occur at higher surface coverage of *O species, leading to the preferential formation of C1 products (formic acid and gaseous CO) on transition metal catalysts,^31^ compared to the precious metals such as Pt and Au.^12,19^ Our results therefore suggest that while oxidized Ni states can activate glycerol oxidation, their abundant availability on heterogeneous catalysts drives selectivity toward C1 products, rather than C3 and C2 products. Such mechanistic insight informs the design of Ni-based catalysts, where strategies to modify interfaces to control surface oxidation (e.g., via electrolyte pH to control the *O species abundancy) and catalyst structure (e.g., catalyst crystallinity, which tunes the distribution/spacing of Ni sites and adsorption energy) can potentially enable the formation of higher valued C3 and C2 products.

**Figure 5:**
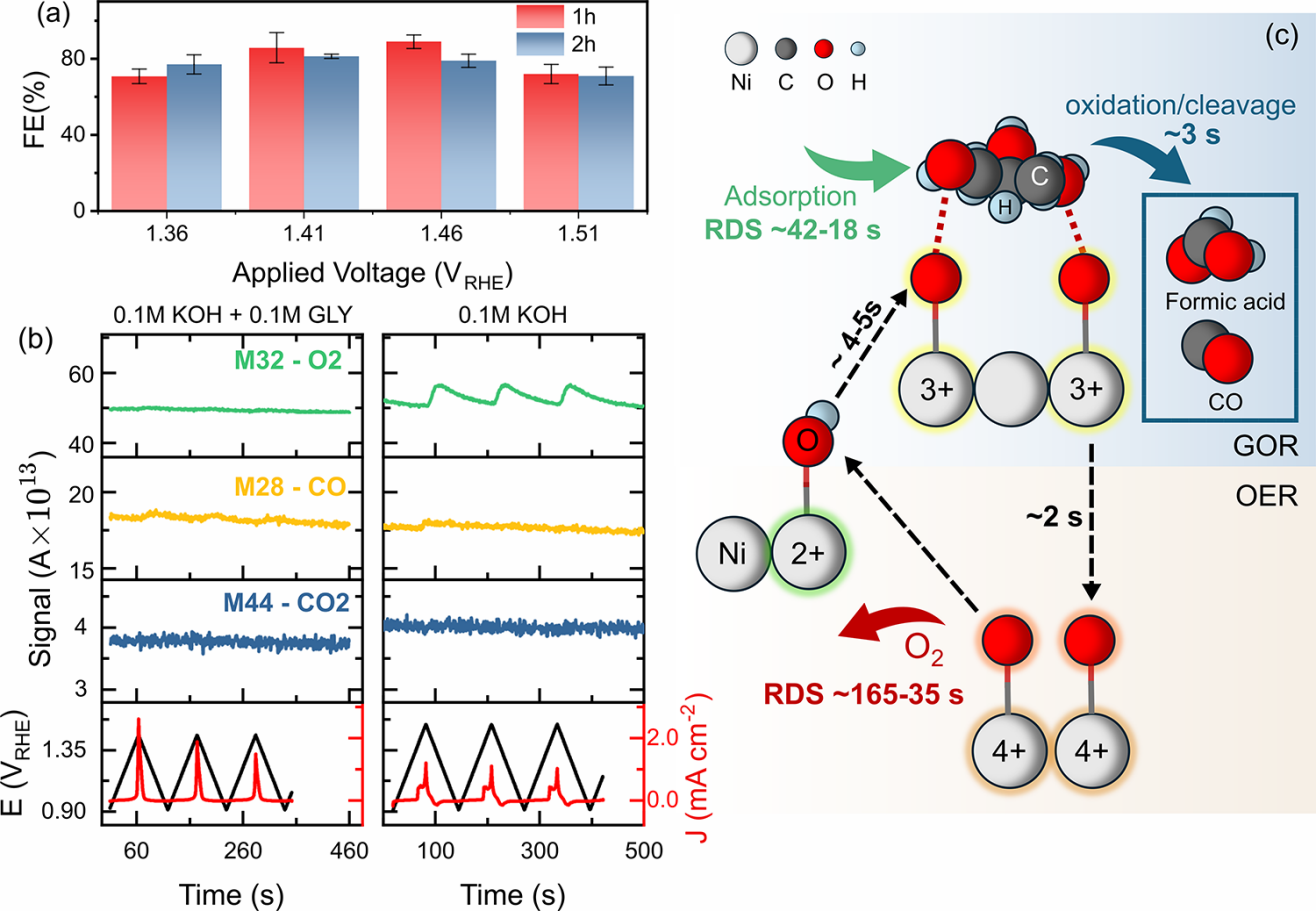
Product analysis and schematics. (a) Faradic efficiency of GOR in 0.1 M KOH + 0.1 M GLY, error bars represent ±σ from two independent measurements at each potential. Note that the only product detected by HPLC was formic acid; thus, the product is not specified in the figure. (b) EC-MS signal for gaseous product detection under 0.1 M KOH + 0.1 M GLY and 0.1 M KOH conditions. Only selected species signal (*m*/*z* = 32, 28, and 44) are displayed here in arb. units. Others showed no obvious change. All measurements used Hg/HgO (0.1 M KOH) as the reference electrode and a Pt coil as the counter electrode. (c) Proposed reaction mechanism and the time constants for each charge-transfer process for GOR and OER.

## Conclusion

In conclusion, we have experimentally identified and quantified the active oxidizing species responsible for the glycerol oxidation reaction (GOR) on Ni­(OH)_2_ catalysts. Operando optical UV–vis coupled with operando NEXAFS were used to monitor the oxidation states of Ni species under operating conditions, where NiOOH (Ni^3+^) was found to be the active species responsible for GOR, while the formation of NiOO (Ni^4+^) species is completely suppressed. Subsequent intrinsic kinetic analysis using time-resolved spectroelectrochemistry showed that the surface adsorption of glycerol molecules is significantly slower than both the formation of Ni^3+^ species and the intrinsic GOR reaction rate, suggesting that the rate-determining step is the accumulation of surface-adsorbed glycerol, in contrast to the observation under the OER condition, where the oxygen evolution is the slowest step. The potential-independent kinetics of glycerol oxidation to formic acid further reveal that the glycerol reaction follows an apparent first-order dependence on the concentration of NiOOH (Ni^3+^) species. Even at potentials >1.55 V_RHE_, the absence of Ni^4+^ states in the presence of glycerol, together with the order of magnitude higher intrinsic reaction kinetics of GOR compared to OER, results in the complete suppression of OER. Overall, these findings suggest the importance of monitoring the potential-dependent oxidation states and probing charge-transfer dynamics using time-resolved spectroscopic techniques, providing a fundamental understanding of the selectivity and reaction mechanism of glycerol oxidation, offering design strategies for Ni-based catalysts, and extending more broadly to the electrocatalytic oxidation of other biomass-derived organic molecules.

## Supplementary Material



## Notes

Materials preparation, additional sample characterization (SEM, XPS, XANES, XRD), operando UV–vis spectroscopy data, stepped-potential spectroelectrochemistry, NEXAFS reference spectrum, HPLC calibration curves, electrochemical measurements and EC-MS (PDF)
