# Supplementary material for: Mechanistic Study of Glycerol Electro-Oxidation on Ni(OH)2/NiOOH Electrodes
Source: J Am Chem Soc. 2026 Apr 7;148(15):16119–28. doi: 10.1021/jacs.6c00726 (PMC13107465; doi:10.1021/jacs.6c00726)
Supplement: Supplementary file 1 [file ja6c00726_si_001.pdf]

Supplementary Information

Mechanistic Study of Glycerol Electro-oxidation on $Ni(OH)_2$/NiOOH electrodes

Youli Yu, [a b] Yifeng Wang, [a] Hanzhi Ye, [c] Sid Halder, [a] Guangmeimei Yang, [d] Boxi Ye, [a] Santosh Kumar, [e] Georg Held, [e] James R. Durrant, [d] Maria-Magdalena Titirici, [c f*] Reshma R. Rao [a b*]

[a] Department of Materials, Imperial College London, Exhibition Road, SW7 2AZ, London

[b] The Grantham Institute for Climate Change, Imperial College London, Exhibition Road, SW7 2AZ, London

[c] Department of Chemical Engineering, Imperial College London, Exhibition Road, SW7 2AZ, London

[d] Department of Chemistry, Imperial College London, Exhibition Road, SW7 2AZ, London

[e] Diamond Light Source, Harwell Science and Innovation Campus, Fermi Ave, OX11 0DE, Didcot

[f] Advanced Institute for Materials Research, Tohoku University, 2 Chome-1-1 Katahira, Aoba Ward, Sendai, Miyagi 980-8577, Japan

Corresponding authors:
m.titirici@imperial.ac.uk (Prof. Maria-Magdalena Titirici)
reshma.rao@imperial.ac.uk (Dr. Reshma R. Rao)

Contents

S1 Materials preparation 3

S2 Materials Characterisation 4

S3 Operando Optical UV-vis Absorption Spectroscopy 7

S4 Operando Near-edge X-ray absorption fine structure spectroscopy (NEXAFS) 12

S5 Intrinsic Kinetic analysis 13

S6 High-performance liquid chromatography (HPLC) 15

S7 Electrochemical measurement 17

S8 Electrochemical Mass Spectroscopy (EC-MS) 18

S1 Materials preparation

$Ni(OH)_2$ films were prepared via electrodeposition. First, a 0.1 M $Ni(NO_3)_2$ hydrate (99.999% trace metal, Merck) solution was prepared. Then a 2-electrode setup was used for electrodeposition, a Pt plate with surface area of 1.5 cm^2 was used as the counter electrode and Hg/HgO (filled with 0.1 M Fe-free KOH) was used as the reference electrode. For the working electrode, different substrates including fluorine-doped tin oxide (FTO, TEC15, Harford) glass substrates; SiN_x window (Silson Ltd.), carbon paper (Freudenberg H23) and glassy carbon (GC, HTW Germany) were adopted for different measurements. An anodic current of -0.1 mA/cm^2 was applied against the Pt foil counter electrode for 600 s (60 mC passed) for the electrodeposition. All substrates were cleaned and rinsed with DI water followed by drying with compressed N_2 gas before and after the electrodeposition.

S2 Materials Characterisation

Scanning electron microscopy (SEM) images were taken on Zeiss Gemini Sigma 300. X-ray photoelectron spectroscopy (XPS) was conducted on *Thermo Fisher K-Alpha+* with a monochromated Al Kα Micro-focused source. A flood gun was used to minimize the sample charging effect. Ex-situ X-ray Absorption Spectroscopy (XAS) of Ni K-edge measurements were taken at B18 beamline at Diamond Light Source. The measurements were collected in fluorescence mode. The resulting spectra were analysed using the software *Larch*. X-ray Diffraction (XRD) measurements were taken on Bruker D2 phaser (Cu Kα).

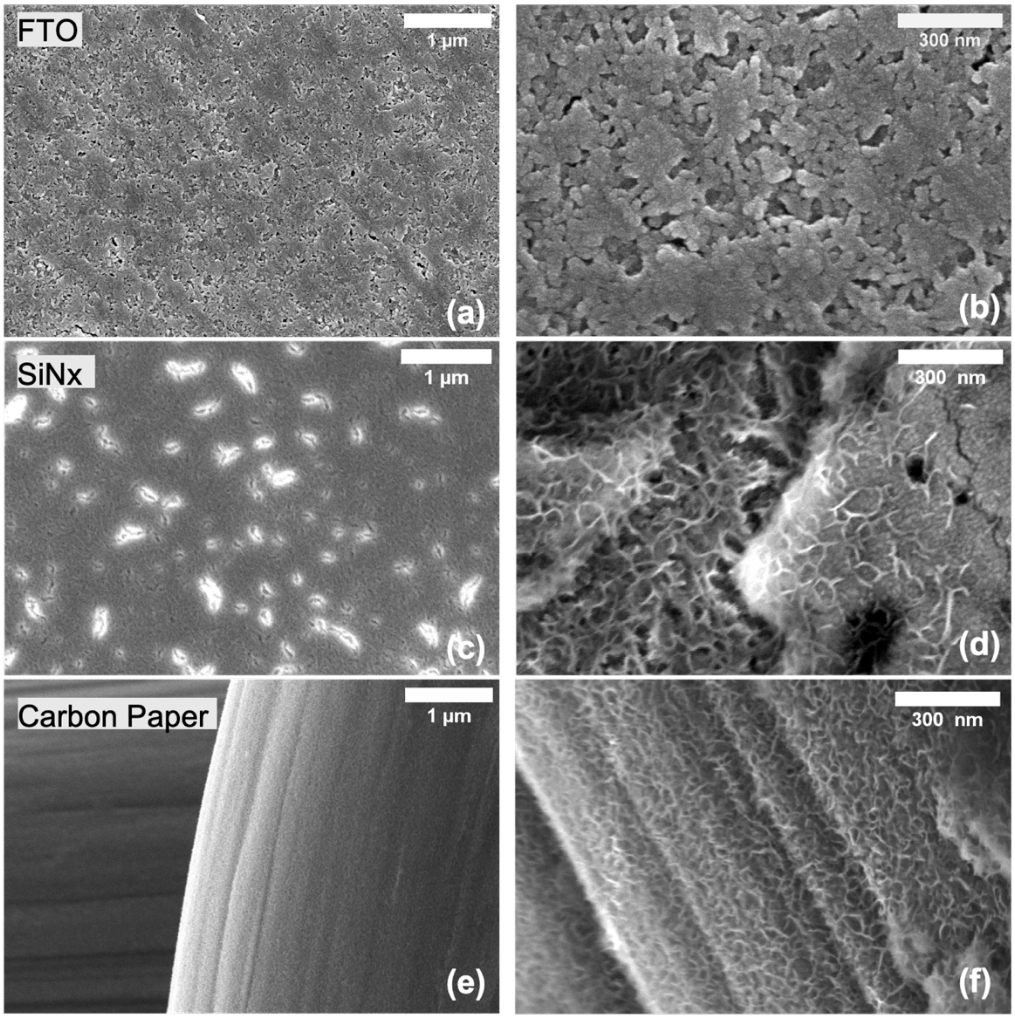

Supplementary Figure 1 SEM images of electrodeposited $Ni(OH)_2$ on FTO (a,b), SiNx (c,d) and carbon paper (e,f,).

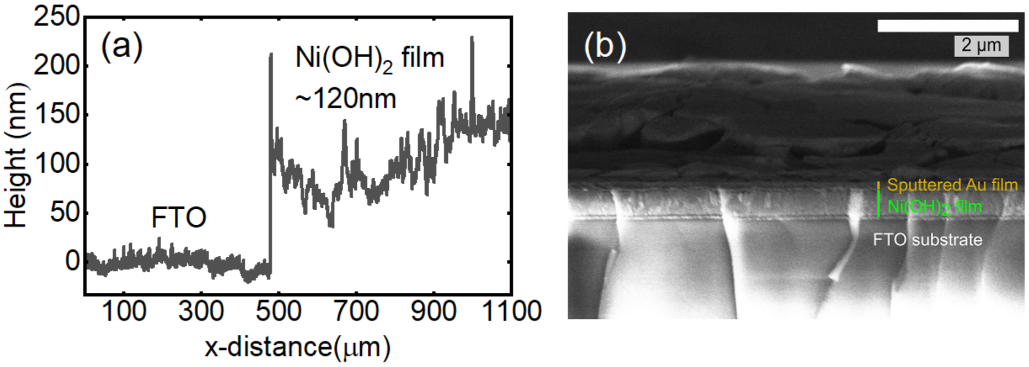

Supplementary Figure 2 (a) Profiler and (b) cross-sectional SEM image of electrodeposited $Ni(OH)_2$ on FTO substrate. The film thickness was measured to be ~120 nm with average roughness (Ra) being 26 nm compared to 6.5 nm for FTO substrate.

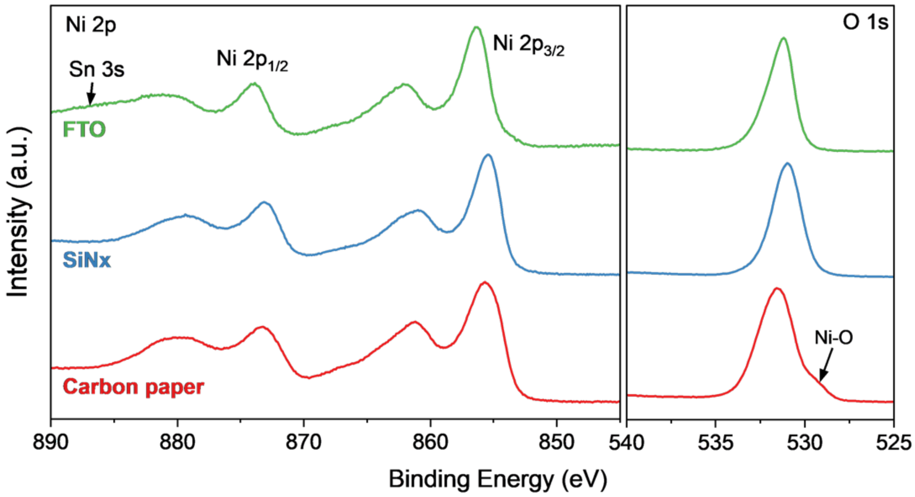

Supplementary Figure 3 XPS spectrum of electrodeposited $Ni(OH)_2$ on FTO, SiNx and carbon paper. The spectra were calibrated against the C 1s spectrum at the binding energy of 284.8 eV.

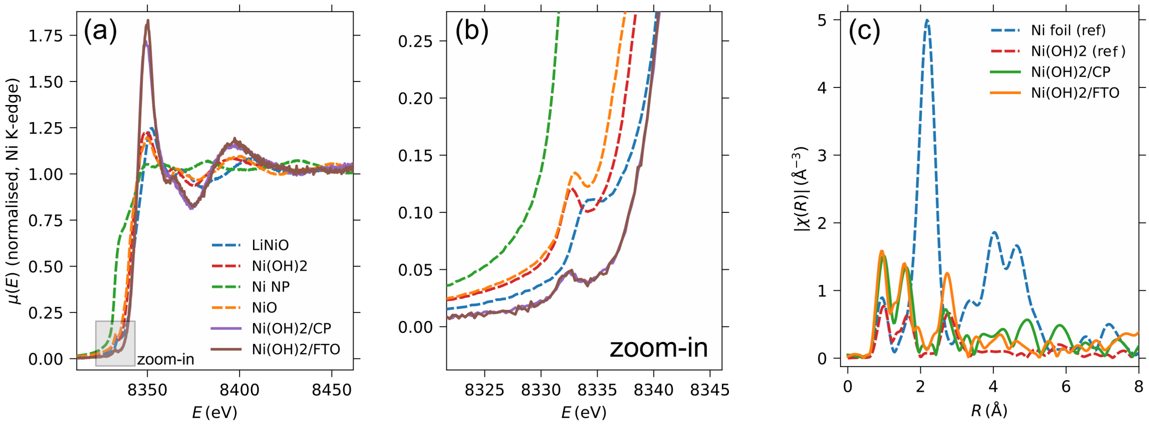

Supplementary Figure 4 (a) Normalized XANES of Ni K-edge for pristine electrodeposited $Ni(OH)_2$ on FTO and carbon paper, (b) zoom-in of the pre-edge feature. (c) k2-weighted Fourier transforms EXAFS spectra collected at Ni K-edge. The energy was calibrated against the standard Ni foil reference sample.

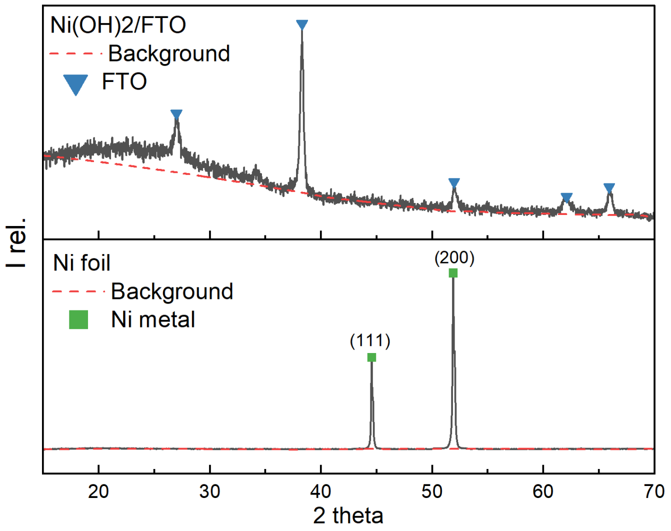

Supplementary Figure 5 XRD patterns of electrodeposited $Ni(OH)_2$ on FTO (FTO peak assignment based on reference[3]) in upper panel and Ni foil in the bottom panel. The XRD pattern was scanned from 15 $\leq 2\theta \leq$ 70 with a step size of 0.1°.

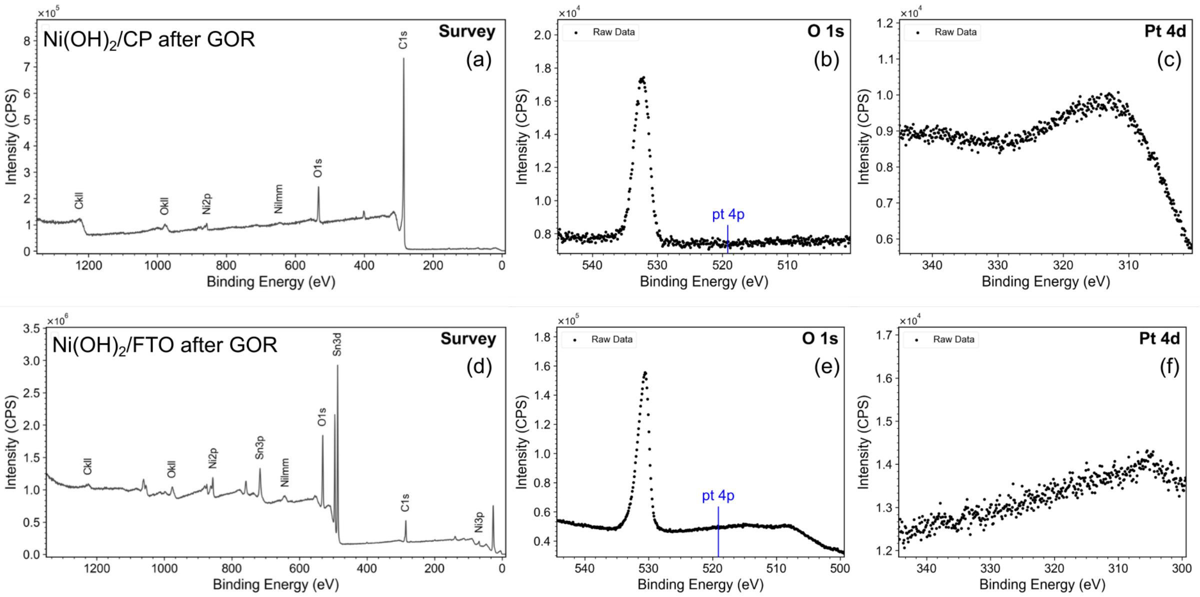

Supplementary Figure 6 XPS spectrum of electrodeposited $Ni(OH)_2$ on carbon paper (a-c) and FTO (d-e) after 10 CV cycles (10 mV/s) in 0.1M KOH + 0.1M GLY using a Pt coil as counter electrode and a Hg/HgO (0.1M KOH) reference electrode.

S3 Operando Optical UV-vis Absorption Spectroscopy

The protocol for operando UV Vis measurements has been outlined in our previous work. [4–9] In short, the measurement was performed in transmittance mode with a home-made one compartment PEEK cell with a quartz window. A 10-mW tungsten-halogen light source (Thorlabs SL201L with SLS201C collimator) was directed through a color balancing filter, then received via a spectrometer (Andor Kymera 193i) coupled with a CCD camera (Andor iDus Du420A-BEX2-DD) to process the transmitted light. The detector was held at -80 C for 2 hours prior to and during the measurement to optimize the signal-to-noise ratio. Samples were prepared by electrodepositing on FTO substrate. The FTO substrate was cleaned following a sequence of sonication in acetone, isopropanol, and DI water for 10 mins each in prior to use. The area for electrodeposition was constrained to be ~1 cm^2 by Kapton tape.

The open-source script used for data analysis in this study can be found on https://github.com/Steven687548/SEC_GUI.git. The step-by-step resulting spectra and calculations will be shown below:

(1) Differential analysis

The optical spectra were recorded by scanning continuously under potentiostatic mode at 1 mV/s. Each measurement took 30 ms, for each 1 mV interval, ~30 scans were taken and averaged. Here all the spectra are plotted in change of absorbance with reference to the starting potential spectrum (0.91 V_{RHE}). To separate the redox transitions across the potential window, the absorption spectra were extracted and compared by performing the differential analysis at 20 mV interval, each identified redox transition should have consistent and distinct spectral feature after normalisation.

0.1M KOH + 0.1M glycerol

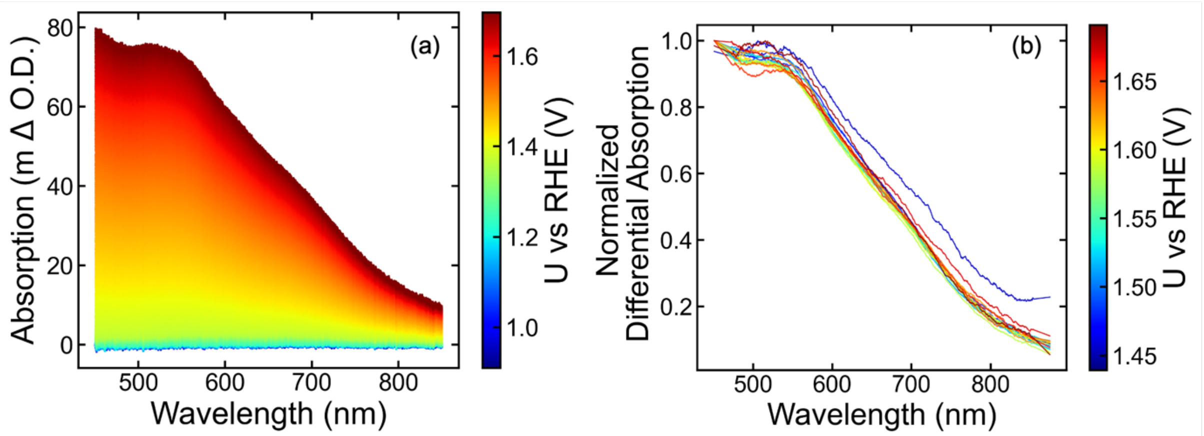

Supplementary Figure 7 (a) Absorption spectra of electrodeposited $Ni(OH)_2$ in 0.1M KOH + 0.1M GLY, scanned from 0.91 to 1.71 V_{RHE} (iR-corrected) in a linear sweep voltammetry at 0.1 mV s^{-1} scan rate. The differential absorption is calculated against the reference spectra at 0.91 V_{RHE}. (b) The differential absorption spectra is collected by subtracting the previous one at an interval of 20 mV and normalize against the maximum absorbance, shows the spectral change upon every 20 mV increase in potential.

<u>0.1M KOH</u>

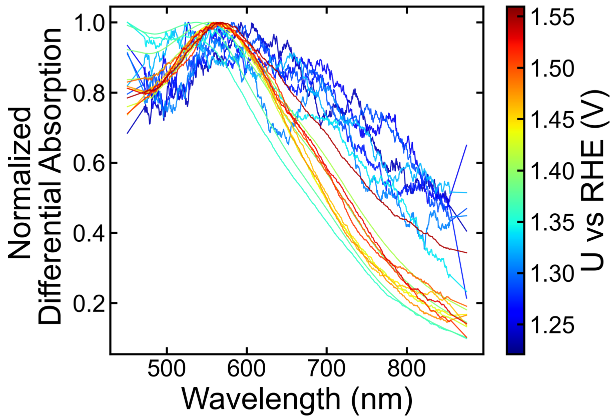

Supplementary Figure 8 Differential analysis of absorption spectra in 0.1 M KOH condition following the same procedure as above. The normalized differential absorption spectra were found to have three distinctive spectral features at an interval of 20 mV.

(2) Linear combination fitting

linear combination fitting was carried out at each species' absorption maxima across its determined potential range. According to the Lambert-Beer's law, the fitted absorption results represent the contribution from each oxidising species in optical signal.

<u>0.1M KOH + 0.1M glycerol</u>

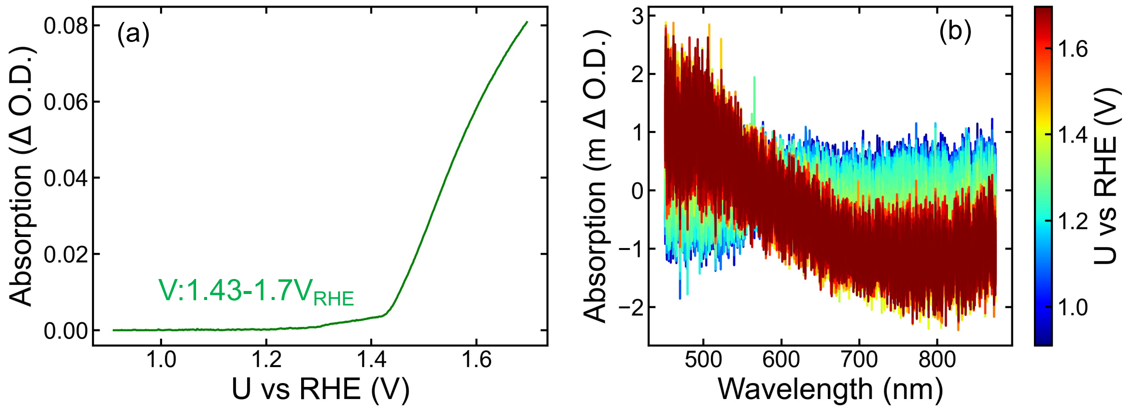

Supplementary Figure 9 (a) Calculated absorption at 550 nm (the absorption maxima correspond to this redox transition) against the potential. (b) Fitting residuals between calculated spectra and the experimental spectra at each potential. The fitting residual was found to be within the range of ±3 m ΔO.D. (within 5% residual range), far below the maximum absorption of around 80 m ΔO.D.

0.1M KOH

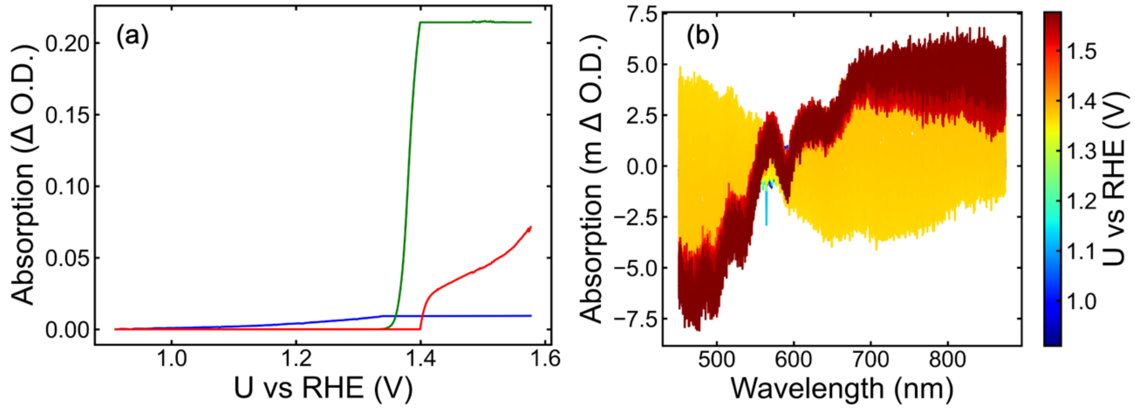

Supplementary Figure 10 (a) Calculated absorption at 550 nm (the absorption maxima correspond to this redox transition) against the potential. (b) Fitting residuals between calculated spectra and the experimental spectra at each potential. The residual is within ± 5% of the optical absorption across the potential. The fitting residual was found to be within the range of ±7.5 m ΔO.D. (within ~5% residual range), far below the maximum absorption of around 275 m ΔO.D.

(3) Extinction coefficient measurement

To convert the optical signal from the linear combination fitting to quantity of charge supplied, we can experimentally measure and calculate the extinction coefficient assume there is linear relationship between the optical absorption and the charge supplied.

$$Extinction\ Coefficient\ (\Delta O.D.C^{-1}cm^{-2}) = \frac{\delta\ \Delta A\ (\lambda)}{\delta Q_i} \qquad (Eq\ S1)$$

By performing square wave analysis, the reductive charge from applying stepped potential was directly plotted against the average of absorption. Specifically, the reductive charge was calculated by integrating the area after applying the stepped potential. The extracted charge per area (Q_i) was then correlated with the average of the maximum optical density when applying the stepped potential ($\Delta A\ (\lambda_{max})$), and the extinction coefficient was fitted and calculated according to the equation above where the y-intercept was constrained to be zero.

0.1M KOH + 0.1M glycerol

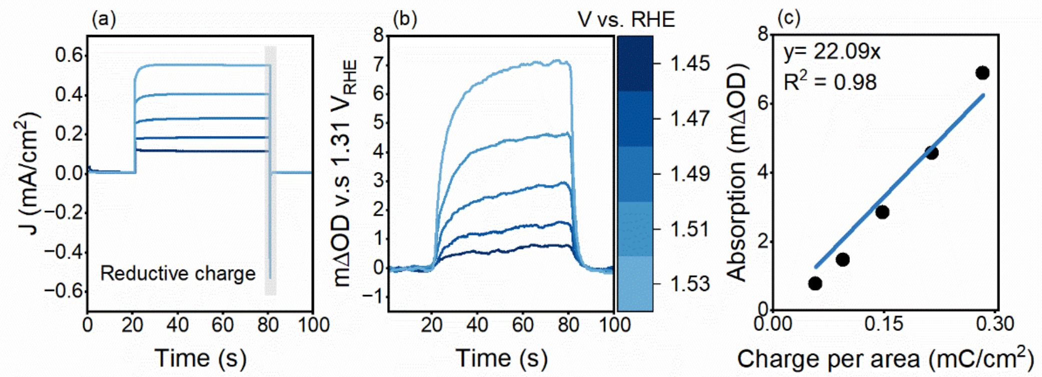

Supplementary Figure 11 The extinction coefficient calculation for $Ni(OH)_2$ to NiOOH redox transition in 0.1M KOH + 0.1M GLY condition. (a) Current response upon applying a stepped potential from 1.31 V_{RHE} to the determined potential in redox transition region, then stepped back to the starting potential. The reductive charge from 80 s onwards is calculated by integrating the area. (b) The smoothed optical signal upon the same stepped potential profile. The average optical absorption value between 22 s to 78 s was extracted. (c) The extracted optical absorption was plotted against the integrated reductive charge, linear fitting (blue line) was performed, extinction coefficient is the slope of the fitted straight line.

0.1M KOH

Three redox transitions have been identified from the differential analysis in 0.1M KOH condition. However, the first transition at 1.2-1.3 V_{RHE} was not concluded in the deconvolution because it contributed to very minor optical signal (~1 m$\Delta O.D.$), and the very narrow potential window (1.34-1.39 V_{RHE}) of second redox transition ($Ni(OH)_2$ to NiOO) makes the measurements less accurate, here we follow the same method from our previous study to estimate extinction coefficient.[4] The third redox transition of NiOOH to NiOO is measured and calculated as shown in Supplementary Figure 12.

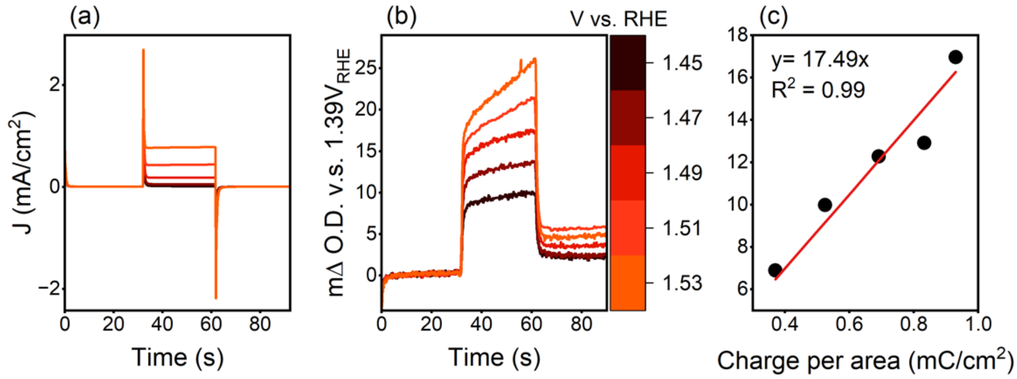

Supplementary Figure 12 The extinction coefficient calculation for the NiOOH to NiOO redox transition in 0.1M KOH condition. (a) Current response upon applying a stepped potential from 1.39 V_{RHE} to the determined potential in redox transition region, then stepped back to the starting potential. The reductive charge from 60s onwards is calculated by integrating the area. (b) The smoothed optical signal upon the same stepped potential profile. The average optical absorption value between 32 s to 58 s was extracted. (c) The extracted optical absorption was plotted against the integrated reductive charge, linear fitting (redline) was performed, extinction coefficient is the slope of the fitted straight line.

(3) Redox transition concentration

The fitted extinction coefficient can then be used to calculate the concentration of the oxidising species by converting the linear fitting of optical absorption across the potential (Supplementary Figure 9,10 (a)) to redox transition concentration per cm^2.

$$[Redox\ caoncentration](C\ cm^{-2}) = \frac{Absorption}{Extinction\ coefficient} \qquad (Eq\ S2)$$

For example, assume each $Ni(OH)_2$ to NiOOH transition requires one electron, the number of charges is equivalent to the concentration of oxidising species ($[NiOOH]$).

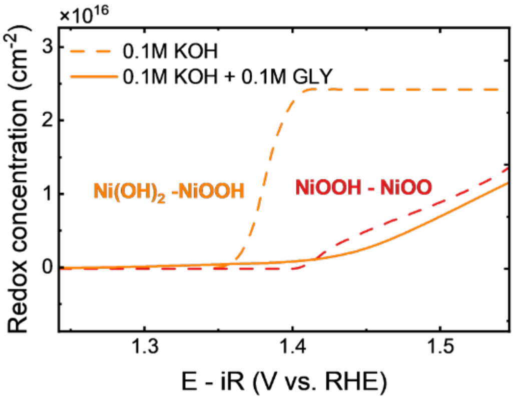

Supplementary Figure 13 Redox concentration of oxidising species under 0.1M KOH (solid) and 0.1M KOH + 0.1M GLY (dotted) condition.

The derivative of the redox concentration can thus be calculated according to the equation below, representing the redox transition waves.

$$redox\ transition\ concentration\ (cm^{-2}V^{-1}) = \frac{\delta\ [Redox\ concentration](C\ cm^{-2})}{\delta\ potential} \times 6.25 \times 10^{18} \qquad (Eq\ S3)$$

S4 Operando Near-edge X-ray absorption fine structure spectroscopy (NEXAFS)

Operando NEXAFS for Ni L-edge and O K-edge was measured at B07 beamline (Branch B) at Diamond Light Synchrotron. A home-made PEEK flow cell with 3-electrode configuration was used, with a Pt rod as counter electrode and a miniature Ag/AgCl (sat. KCl) as the reference electrode, the detailed in-situ flow cell design is published.[10] The samples were electrodeposited on a 100 nm thick SiN_x window (*Silson* Ltd.) coated with 10nm Ti (adhesive layer) and 10nm Au (conductive layer). The SiN_x window enables high transmission of soft x-ray towards the tested catalyst and serves as a separator between the vacuum chamber (~10^{-4} mbar) and the ambient pressure condition inside the cell. For data analysis, a four-step protocol was followed: (1) the data were normalised against the I_0 (indicent photon flux) to correct for beamline intensity fluctuations; (2) a line fitted to the pre-edge region was subtracted to remove the background; (3) the data were then normalized to the average post-edge value; (4) energy calibration was performed by referring to the O K-edge standard.

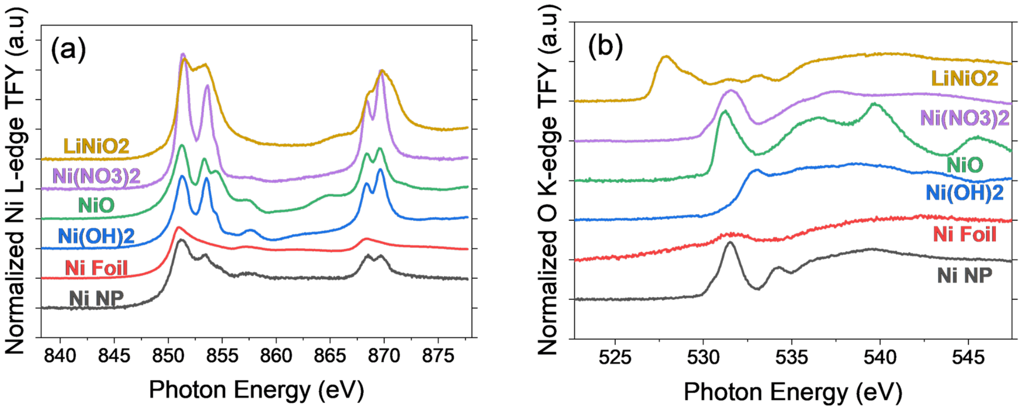

Supplementary Figure 14 Normalized Ni L-edge (a) and O K- edge (b) of reference spectrum collected in total florosence mode, ranging from $LiNiO_2$ (Ni^{3+}) to Ni NP.

S5 Intrinsic Kinetic analysis

Intrinsic kinetic analysis can be performed by initial rate linear fitting upon a change in potential was applied. Specifically, the potential profile is operated as: first applying a potential prior to the OER/GOR potential region for 20 s, then stepping to a higher potential where GOR/OER happening until the optical signal stabilise; afterwards, the potential was released to OCP while monitoring the optical signal. The decay of absorption can be used to evaluate the kinetic time constant of each charge transfer process, as described in detail in the main text.

<u>0.1M KOH + 0.1M GLY</u>

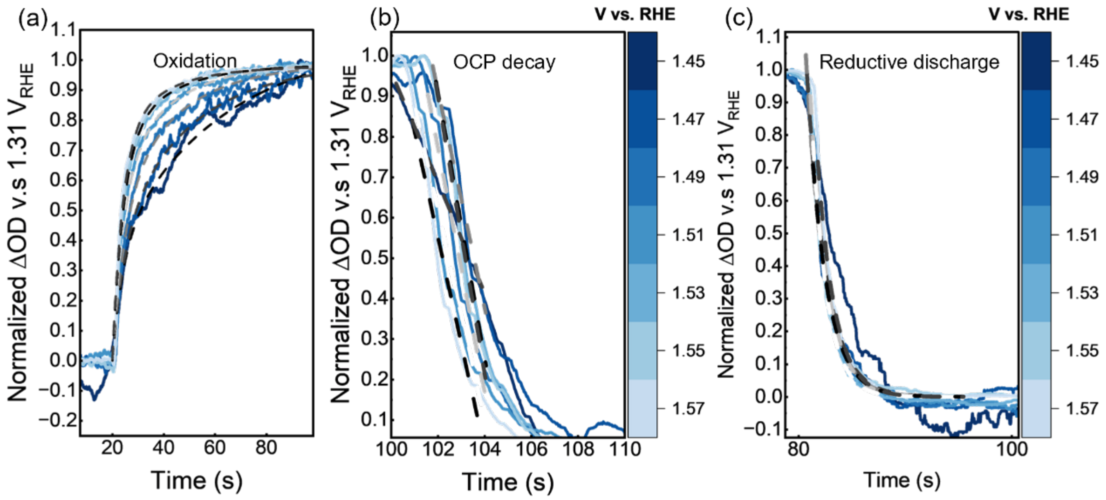

Supplementary Figure 15 Time-resolved optical signal at 550 nm under 0.1M KOH + 0.1M GLY condition for stepped potential decay analysis. (a) Smoothed optical signal upon OCP decay measurement. Normalized exponential grow fitting for (b) charge accumulation step and initial rate for (c) OCP decay step.

Supplementary Table 1 Summary of the time constant fitting parameters and result under 0.1M KOH + 0.1M GLY condition.

Applied potential (V v.s. RHE)	A1 (0-1)	τ1 (s)	A2 (0-1)	τ2 (s)	τ3 – decay (s)	τ4 – reductive discharge(s)
1.45	0.36	4.25	0.70	41.76	3.67	-
1.47	0.37	4.55	0.61	27.89	3.16	2.04
1.49	0.50	5.02	0.49	23.45	3.18	1.87
1.51	0.58	4.19	0.41	20.21	3.26	1.74
1.53	0.68	4.56	0.30	18.03	2.95	1.49
1.55	0.71	4.31	0.28	19.93	2.82	1.75
1.57	0.74	4.03	0.24	18.47	3.30	1.59

0.1M KOH

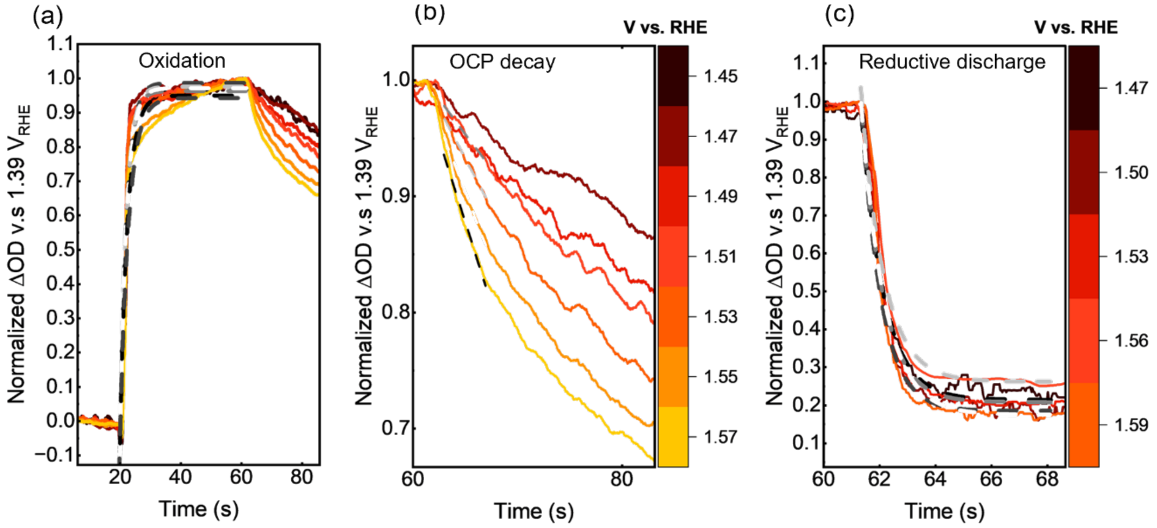

Supplementary Figure 16 Time-resolved optical signal at 567 nm under 0.1M KOH condition for stepped potential decay analysis. (a) Smoothed optical signal upon OCP decay measurement. Normalized exponential grow fitting for (b) charge accumulation step and initial rate for (c) OCP decay step.

Supplementary Table 2 Summary of the time constant fitting parameters and result under 0.1M KOH condition.

Applied potential (V v.s. RHE)	τ1- oxidation (s)	τ3 – decay (s)	τ4 – reductive discharge (s)
1.47	2.20	165.42	1.85
1.49	2.07	115.46	1.75
1.51	2.09	83.66	1.63
1.53	2.19	58.33	1.42
1.55	2.51	43.14	1.75
1.57	2.88	35.37	1.62

S6 High-performance liquid chromatography (HPLC)

HPLC was performed with an Agilent 1260 Infinity II LC system. A Biorad HPX-87H column was kept at 40 °C and coupled with 2 detectors: an RID at 35°C and a VWD at 210nm to avoid the peak-overlap issues at close retention times. HPLC was calibrated against different concentrations of known compounds, establishing a linear relationship between the integrated area and the known concentrations as shown in Supplementary Figure 17. The mobile phase was 5 mM H_2SO_4 with a flow rate of 0.2 mL/min, and the injection volume of 1 µL. The samples were electrodeposited on the carbon paper (Freudenberg H23) for chronoamperometry (CA) measurement. For each measurement, 10mL 0.1M iron-free KOH + 0.1M GLY was used, and the solution was collected at 1- and 2-hour interval at constant potential of 1.36, 1.41, 1.46 and 1.51 V_{RHE}. A magnetic stirring bar was used during the reaction. 200 µL sample solution was immediately acidified with 20µL 0.55M H_2SO_4 and 180µL DI water after taking out from the cell, to prevent further reaction under alkaline conditions. The Faradaic Efficiency (FE) was calculated through the following equation:

$$FE(\%) = \frac{n_{product\ formed} \times zF}{Q} \times 100\% \qquad (Eq\ S4)$$

Where n is the mole quantity of the formed product, z is the number of electrons required for the product to form, F is the Faraday constant, Q is the charge passed. In this study, the only product detected was formic acid, which requires 8/3 mol of electrons to produce 1 mol formic acid from glycerol as shown below:

$$C_3H_8O_3 + 3H_2O = 3HCOOH + 8e^- + 8H^+$$

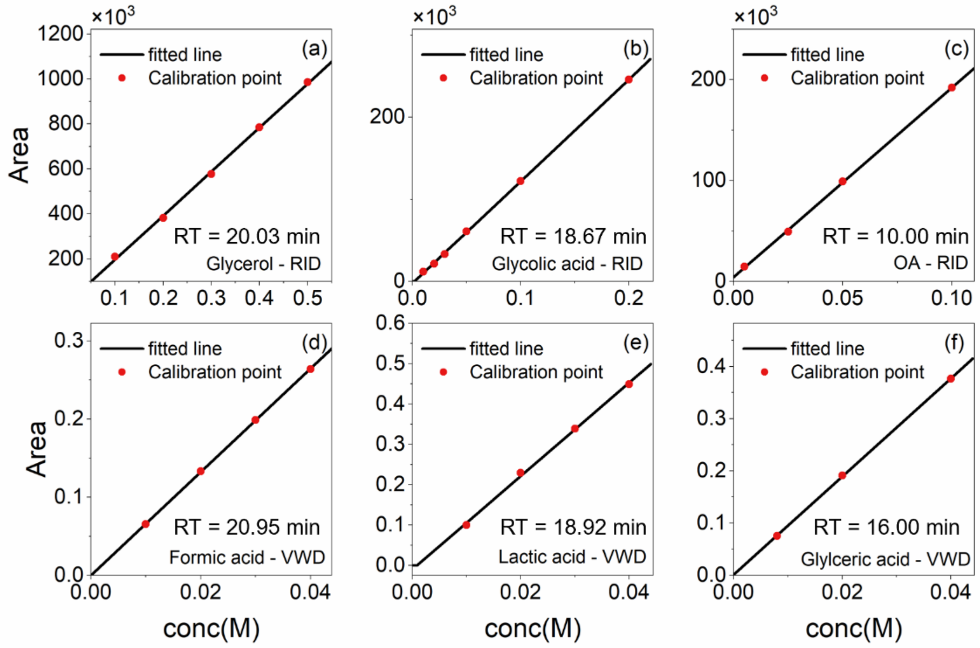

Supplementary Figure 17 HPLC calibration curve of 6 main compounds under different concentrations, separated using 2 detectors. (a) Glycerol, (b) Glycolic acid, (c) Oxalic acid with RID detector and (d) Formic acid, (e) Lactic acid, (f) Glyceric acid with VWD detector.

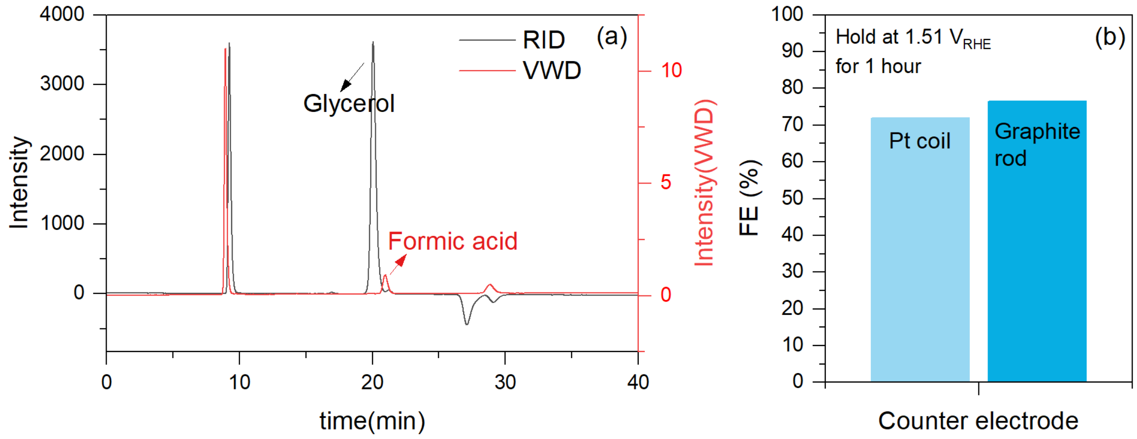

Supplementary Figure 18 (a) Example of HPLC signal from RID (black) and VWD (red) detectors, where the reaction has been held at 1.51 V_{RHE} for 2 hours, only formic acid and glycerol was detected. (b) Faradaic efficiency of comparing Pt coil and graphite rod as counter electrode during GOR at 1.51 V_{RHE} for 1 hour, formic acid was the only product detected by HPLC under these conditions.

S7 Electrochemical measurement

The electrochemical test was conducted in a 3-electrode set-up with a 30 mL PTFE beaker using an Autolab potentiostat, with a Pt coil as the counter electrode and a Hg/HgO (0.1 M Fe-free KOH) as the reference electrode. The electrochemical measurements were performed in iron-free 0.1 M KOH (suprapur, ≥99.995%, Merck) and 0.1 M KOH + 0.1 M glycerol (>99.5%, Merck). The electrolyte was purged with N_2 gas for 30 mins prior to each measurement.

Note that all the 0.1 M KOH electrolytes were purified following the protocol of Trotochaud et al. to exclude trace Fe impurity that could distort the results. [1,11–13] Briefly, 2 g of $Ni(NO_3)_2 \cdot 6H_2O$ were dissolved in 4 mL of ultra-pure water, then mixed with 20 ml of 1 M suprapur KOH. The high purity $Ni(OH)_2$ precipitate was collected by centrifuging at 3500 rpm for 3 mins. The precipitate then underwent three redispersion and centrifuging washing cycles with 20 mL ultra-pure water and 2 mL suprapur 1 M KOH. Afterwards, the precipitate was mixed with 45 mL of suparpur 1 M KOH, sonicated for 20 minutes, and left to rest overnight. The resting mixture was centrifuged at 7000 rpm for 25 min, and the purified 1M KOH was collected by syringe-filtering the supernatant. Ultrapure water (18.2 MΩ) was used throughout the project.

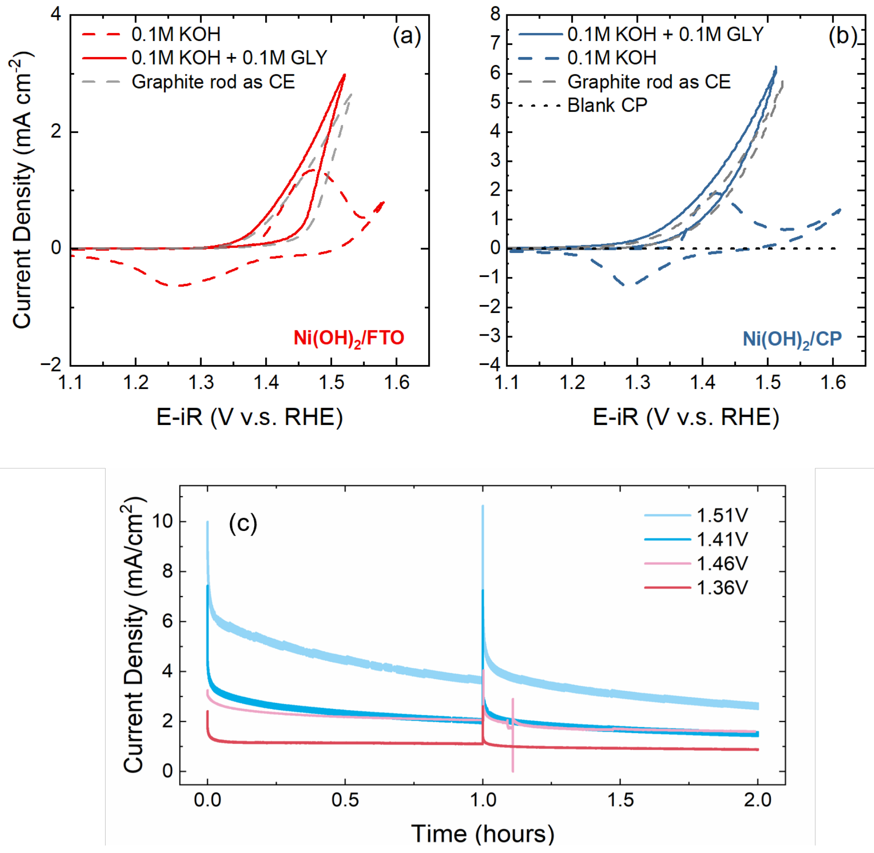

Supplementary Figure 19 CV and CA. 5th cycle of CV scans of electrodeposited $Ni(OH)_2$ on (a) FTO and (b) carbon paper (CP), in 0.1 M KOH (dashed line) and 0.1 M KOH + 0.1 M GLY (solid line), respectively. The blank measurement for carbon paper in 0.1M KOH + 0.1M GLY is shown as dashed black line. The comparison data of using graphite rod as counter electrode in 0.1M KOH + 0.1M GLY is shown as dashed grey line. Scan rate: 10 mV/s. (c) Chronoamperometry (CA) for $Ni(OH)_2$ on CP in 0.1M KOH + 0.1 M GLY at four selected potentials (1.36, 1.41, 1,46, 1.51 V_{RHE}). Noted that Hg/HgO (0.1M KOH) was used as the reference electrode and a Pt coil was used as the counter electrode.

S8 Electrochemical Mass Spectroscopy (EC-MS)

EC-MS was performed at a Spectro-inlets system using a custom designed cell with three-electrode configuration. A nanofabricated silicon chip was used to allow the generated gas species to pass below and be transported to the mass spectrometer by the carrier gas. Freshly polished glassy carbon RDE electrodes with 5 mm diameter were used as the electrodeposition substrate.

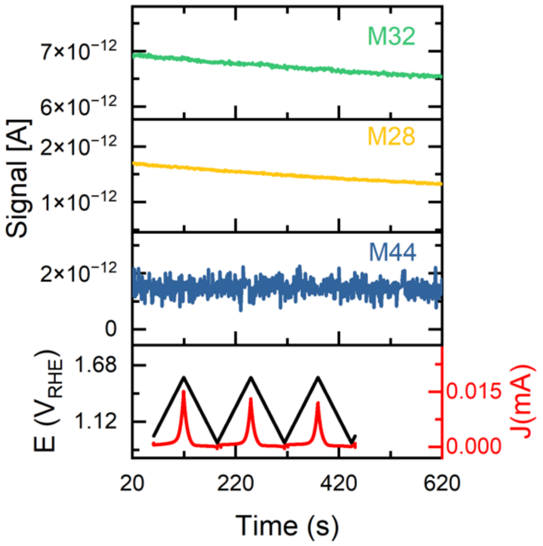

Supplementary Figure 20 ECMS signal for blank glassy carbon in 0.1M KOH + 0.1M GLY condition. No gaseous species were detected under this condition, confirming the authenticity of the detected gaseous species are coming from the catalytic reaction between glycerol and the electrodeposited catalyst rather than the glassy carbon substrate.

S9 Rate law analysis in <u>0.1M KOH + 0.1M GLY</u>

The reaction order can be estimated based on several assumptions: (1) the faradaic efficiency of electrodeposition is 100%, the theoretical maximum catalyst loading can be used; (2) the oxidation current can be obtained by subtracting the charging current from the measured current because the major contributions to the measured current is charging current (for redox transition) and the oxidation current (GOR or OER depends on the electrolyte condition); (3) the electrodeposited film is bulk active. The reaction order can be obtained by performing linear fitting for the glycerol oxidation current against the concentration of NiOOH species, and the slope was found to be ~1.

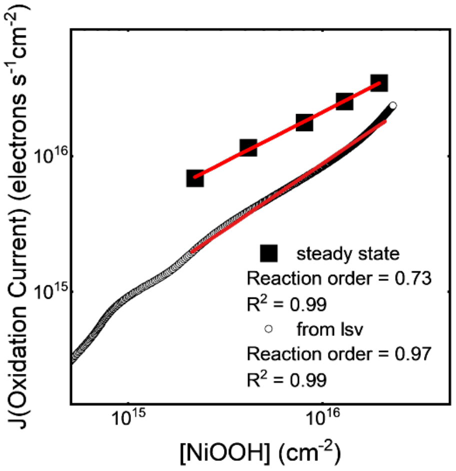

Supplementary Figure 21 Rate law analysis under 0.1M KOH + 0.1M GLY condition.

References

(1) Trotochaud, L.; Young, S. L.; Ranney, J. K.; Boettcher, S. W. Nickel–Iron Oxyhydroxide Oxygen-Evolution Electrocatalysts: The Role of Intentional and Incidental Iron Incorporation. *J. Am. Chem. Soc.* **2014**, *136* (18), 6744–6753. https://doi.org/10.1021/ja502379c.

(2) Francàs, L.; Corby, S.; Selim, S.; Lee, D.; Mesa, C. A.; Godin, R.; Pastor, E.; Stephens, I. E. L.; Choi, K.-S.; Durrant, J. R. Spectroelectrochemical Study of Water Oxidation on Nickel and Iron Oxyhydroxide Electrocatalysts. *Nat. Commun.* **2019**, *10* (1), 5208. https://doi.org/10.1038/s41467-019-13061-0.

(3) Iyengar, P.; Das, C.; Balasubramaniam, K. R. Photoelectrochemical Performance of NiO-Coated ZnO–CdS Core-Shell Photoanode. *J. Phys. D Appl. Phys.* **2017**, *50* (10), 10LT01. https://doi.org/10.1088/1361-6463/aa5875.

(4) Wang, Y.; Twight, L.; Sagui, N.; Kwak, M.; Boettcher, S.; Moss, B.; Stephens, I.; Durrant, J.; Rao, R. Spectroelectrochemical Studies of Oxygen Evolution Reaction Kinetics for Surface Incorporated Iron in Nickel Oxyhydroxide Electrocatalysts. *ChemRxiv*. July 13, 2025. https://doi.org/10.26434/chemrxiv-2025-94ds9-v3.

(5) Moss, B.; Svane, K. L.; Nieto-Castro, D.; Rao, R. R.; Scott, S. B.; Tseng, C.; Sachs, M.; Pennathur, A.; Liang, C.; Oldham, L. I.; Mazzolini, E.; Jurado, L.; Sankar, G.; Parry, S.; Celorrio, V.; Dawlaty, J. M.; Rossmeisl, J.; Galán-Mascarós, J. R.; Stephens, I. E. L.; Durrant, J. R. Cooperative Effects Drive Water Oxidation Catalysis in Cobalt Electrocatalysts through the Destabilization of Intermediates. *J. Am. Chem. Soc.* **2024**, *146* (13), 8915–8927. https://doi.org/10.1021/jacs.3c11651.

(6) Liang, C.; Garcia Verga, L.; Moss, B.; Kumar, S.; Scott, S. B.; Turner, M. A.; Ferrer, P.; Celorrio, V.; Grinter, D. C.; Tao, Y.; Halder, S.; Wang, Y.; Tseng, C.; Yang, G.; Held, G.; Haigh, S. J.; Walsh, A.; Stephens, I. E. L.; Durrant, J. R.; Rao, R. R. Key Role of Oxidizing Species Driving Water Oxidation Revealed by Time-Resolved Optical and X-Ray Spectroscopies. *Nat. Mater.* **2026**. https://doi.org/10.1038/s41563-026-02514-9.

(7) Liang, C.; Katayama, Y.; Tao, Y.; Morinaga, A.; Moss, B.; Celorrio, V.; Ryan, M.; Stephens, I. E. L.; Durrant, J. R.; Rao, R. R. Role of Electrolyte PH on Water Oxidation for Iridium Oxides. *J. Am. Chem. Soc.* **2024**, *146* (13), 8928–8938. https://doi.org/10.1021/jacs.3c12011.

(8) Rao, R. R.; Corby, S.; Bucci, A.; García-Tecedor, M.; Mesa, C. A.; Rossmeisl, J.; Giménez, S.; Lloret-Fillol, J.; Stephens, I. E. L.; Durrant, J. R. Spectroelectrochemical Analysis of the Water Oxidation Mechanism on Doped Nickel Oxides. *J. Am. Chem. Soc.* **2022**, *144* (17), 7622–7633. https://doi.org/10.1021/jacs.1c08152.

(9) Liang, C.; Rao, R. R.; Svane, K. L.; Hadden, J. H. L.; Moss, B.; Scott, S. B.; Sachs, M.; Murawski, J.; Frandsen, A. M.; Riley, D. J.; Ryan, M. P.; Rossmeisl, J.; Durrant, J. R.; Stephens, I. E. L. Unravelling the Effects of Active Site Density and Energetics on the Water Oxidation Activity of Iridium Oxides. *Nat. Catal.* **2024**, *7* (7), 763–775. https://doi.org/10.1038/s41929-024-01168-7.

(10) Kumar, S.; Counter, J. J. C.; Grinter, D. C.; Spronsen, M. A. Van; Ferrer, P.; Large, A.; Orzech, M. W.; Jerzy Wojcik, P.; Held, G. An Electrochemical Flow Cell for Operando XPS and NEXAFS Investigation of Solid–Liquid Interfaces. *Journal of Physics: Energy* **2024**, *6* (3), 036001. https://doi.org/10.1088/2515-7655/ad54ee.

(11) Crago, C. F.; Li, S.; Aleman, A. M.; Siboonruang, T.; Rojas Mendoza, M.; Jaramillo, T. F.; Stevens, M. B. Effects of Iron Impurities and Content on Electrochemical Performance and Oxygen Evolution Selectivity of Nickel Catalysts for Ethanol Oxidation. *J. Am. Chem. Soc.* **2025**, *147* (5), 3925–3930. https://doi.org/10.1021/jacs.4c15365.

(12) Kim, D.; Dang Van, C.; Lee, M. S.; Kim, M.; Lee, M. H.; Oh, J. Selective Formic Acid Production in Ni and NiFe Layered Hydroxides via Glycerol Electro-Oxidation. *ACS Catal.* **2024**, *14* (10), 7717–7725. https://doi.org/10.1021/acscatal.4c01250.

(13) Santana, C. S.; Gjonaj, E.; Garcia, A. C. Effect of Iron Impurities on the Electrochemical Oxidation of Glycerol on $Ni(OH)_2$ /NiOOH Electrodes. *ChemElectroChem* **2024**, *11* (1). https://doi.org/10.1002/celc.202300570.
